# Engineering polysaccharide nanoplatforms for glioblastoma theranostics: Bridging targeted therapy and advanced imaging

**DOI:** 10.7150/thno.123550

**Published:** 2026-01-14

**Authors:** Xiaoming Wang, Qing Yang, Shenglan You, Lei Wu, Qiyong Gong, Yujun Zeng

**Affiliations:** 1 Department of Radiology, Institution of Radiology and Medical Imaging, Huaxi MR Research Center (HMRRC), Frontiers Science Center for Disease-Related Molecular Network, National Clinical Research Center for Geriatrics, State Key Laboratory of Biotherapy, West China Hospital, Sichuan University, Chengdu 610041, China.; 2 Psychoradiology Key Laboratory of Sichuan Province, West China Hospital, Sichuan University, Research Unit of Psychoradiology, Chinese Academy of Medical Sciences, and Key Laboratory of Transplant Engineering and Immunology, NHC, Chengdu 610041, China.; 3 Core Facility of West China Hospital, Sichuan University, Chengdu 610041, China.; 4 Animal Imaging Core Facilities, West China Hospital, Sichuan University, China.; 5 Xiamen Key Lab of Psychoradiology and Neuromodulation, Department of Radiology, West China Xiamen Hospital of Sichuan University, Xiamen 361021, China.

**Keywords:** GBM, imaging, nanomedicines, polysaccharide polymer

## Abstract

Glioblastoma (GBM) is an aggressive brain tumor characterized by limited therapeutic efficacy and challenges in accurate imaging, largely due to its invasive growth, drug resistance, and the restrictive blood-brain barrier (BBB) hindering the delivery of both therapeutic and diagnostic agents. Current GBM treatments and imaging approaches often suffer from insufficient agent penetration into the tumor. Additionally, they frequently exhibit toxicity or poor signal-to-noise ratios. Polysaccharide (PSC)-based polymers, with their inherent biocompatibility, biodegradability, and versatile chemical modifiability, offer a promising platform to overcome these limitations. These natural polymers can be engineered into sophisticated nanocarriers that enhance BBB traversal, enable targeted tumor accumulation of therapeutic payloads and imaging agents Furthermore, they facilitate controlled drug release and improve diagnostic signal generation. Consequently, PSC-based systems can improve therapeutic efficacy and enhance diagnostic accuracy for tumor visualization. Furthermore, they reduce systemic side effects and support multimodal strategies, ranging from single-modality interventions to integrated theranostic systems. This review aims to comprehensively discuss recent advancements, current challenges, and future perspectives of PSC-based nanomedicines in GBM therapy and imaging.

## 1. Introduction

**Figure 1:**
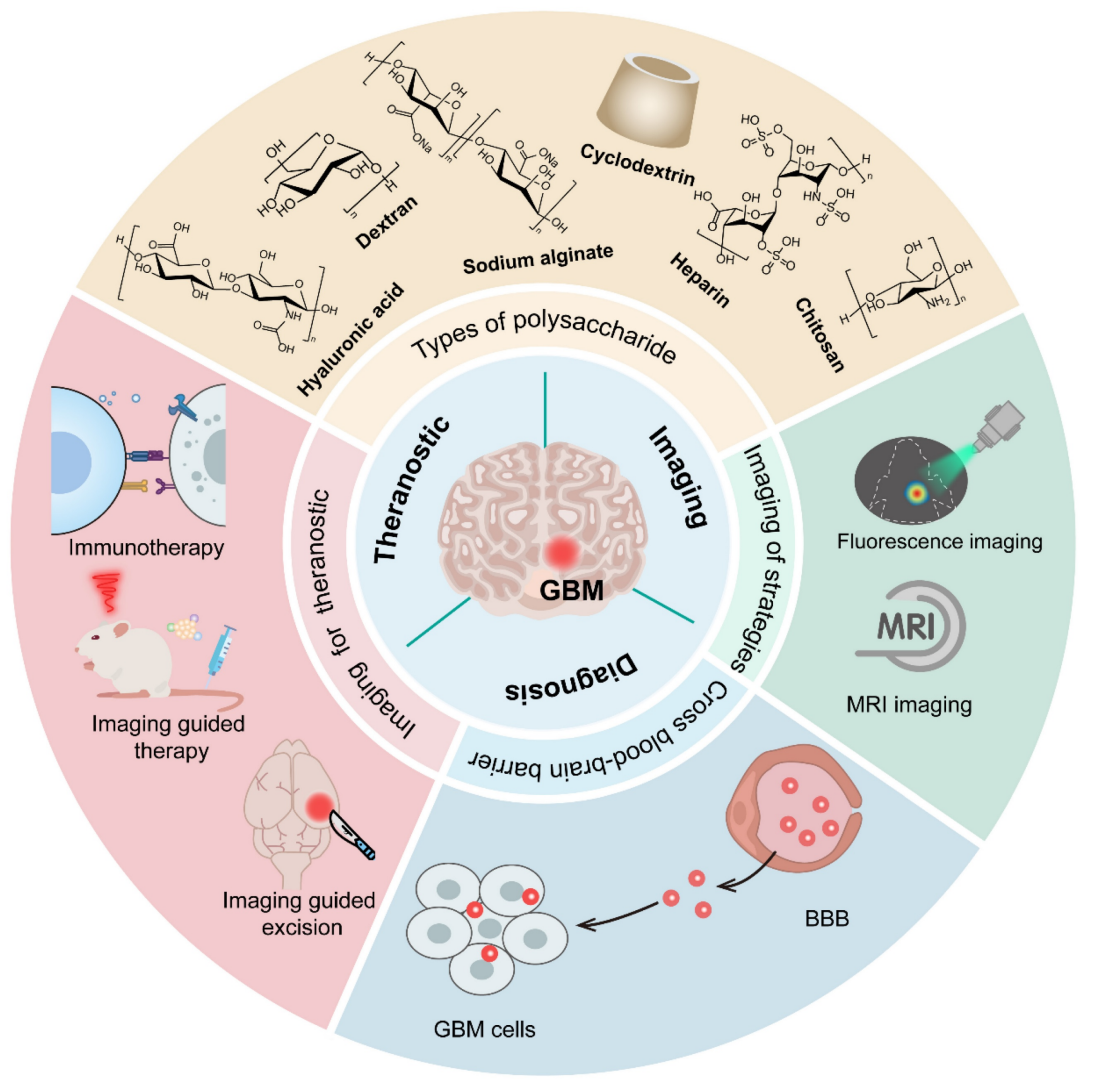
Smart PSC-based nanomedicines (e.g., sodium alginate, chitosan, cyclodextrin, heparin, dextran, etc.) for imaging and therapeutic applications in GBM. Mainly involves (1) crossing the BBB for enhanced accumulation of therapeutic agents and imaging probes at GBM sites; (2) multimodal imaging, including fluorescence imaging and MRI, to support tumor staging, diagnosis, and boundary delineation; (3) theranostic integration, enabling imaging-guided precise treatments (e.g., surgical resection) and immunotherapy.

GBM presents a formidable clinical challenge due to its invasive growth, inherent resistance to standard therapies, and the complex physiological obstacle posed by the BBB 1 (Figure 1). In high-grade gliomas, the BBB evolves into a heterogeneous blood-brain tumor barrier (BBTB), the integrity of which is a subject of considerable debate and a critical factor for drug delivery. This complexity necessitates a clear distinction between the penetration capabilities of small diagnostic agents versus larger therapeutic nanocarriers. For instance, diagnostic agents like gadolinium-based contrast media or ultra-small imaging nanoparticles (NPs) (e.g., <5 nm) can exploit localized structural compromises in the BBTB. The contrast enhancement observed in *T*_1_-weighted magnetic resonance imaging (MRI) directly visualizes regions where the barrier has ruptured, confirming extravasation into the tumor core. However, the level of compromise sufficient for this enhancement is often insufficient for the robust, uniform penetration of larger therapeutic NPs, which typically exceed 50 nm.

The extent of BBB compromise in GBM is highly variable. While high-grade gliomas are characterized by neo-angiogenesis and a "leaky" vasculature in the tumor core-driven by elevated vascular endothelial growth factor (VEGF) and reduced tight junction (TJ) proteins-this breakdown is not uniform. Conflicting studies reveal that vessels in the peritumoral region, where recurrence often originates, frequently retain structural components resembling the healthy BBB, such as intact TJs and normal pericyte coverage. This heterogeneity gives rise to the concept of the 'residual BBTB,' which refers to the anatomical and functional regions within and surrounding the malignant tissue, particularly at the invasive margins, where barrier integrity remains partially or fully intact. This residual barrier, composed of tightly controlled endothelial TJs, astrocyte end-feet, and the basal lamina, actively shields infiltrating tumor cells from systemic therapeutics. The presence of this residual BBTB severely restricts the reliability of the Enhanced Permeability and Retention (EPR) effect for GBM therapy. While the passive accumulation of NPs via the EPR effect can occur in the highly disrupted tumor core, it is largely ineffective at the tumor rim, preventing therapeutic agents from reaching the most invasive cell populations. Therefore, reliance on passive delivery is generally insufficient for GBM eradication. Overcoming these obstacles-including inadequate transport across the residual BBTB, poor targeting specificity, and systemic toxicity of potent agents is crucial. This necessitates the development of sophisticated carrier systems capable of precise tumor targeting and controlled payload release.

PSC-based nanoplatforms have emerged as highly promising candidates for addressing these multifaceted challenges in GBM management. PSCs, including widely studied examples like chitosan, hyaluronic acid (HA), dextran, and alginate, as well as various plant-derived PSCs, offer a unique combination of inherent biocompatibility, biodegradability, and abundant reactive groups for versatile chemical modification 2. These characteristics allow for the rational design of PSC-based carriers that can be functionalized with targeting ligands (e.g., peptides, antibodies) or engineered to respond to specific stimuli within the tumor microenvironment. Such modifications facilitate improved specificity of agent accumulation in GBM cells and enable controlled release of diagnostic or therapeutic payloads. Through their tunable physicochemical properties, PSC-based systems serve as versatile platforms that can be tailored for distinct applications, either as dedicated diagnostic probes or therapeutic carriers 3. Beyond these single-modality functions, advanced engineering allows for the integration of both imaging agents and therapeutic payloads into a single construct, thereby actualizing true theranostic approaches 4, 5. It is critical to distinguish these integrated theranostic systems, which simultaneously deliver therapeutic payloads and diagnostic signals, from nanocarriers designed exclusively for a single modality. While single-function systems optimize either sensitivity (imaging) or payload capacity (therapy), true theranostic platforms must balance both, often requiring more complex engineering to prevent signal interference or premature drug release. Consequently, these nanoformulations hold the potential to significantly improve diagnostic accuracy and therapeutic outcomes while minimizing off-target effects. Furthermore, beyond their utility as delivery vehicles, many PSCs possess inherent immunostimulatory properties or can be modified to modulate the tumor immune microenvironment, offering an additional avenue to enhance anti-GBM responses 6.

Given the significant advantages and versatile nature of PSC nanoplatforms in addressing the critical issues faced in GBM diagnosis and treatment, this review aims to comprehensively summarize the recent advancements in the field. We will delve into the various types of PSCs being explored, such as chitosan, HA, dextran, alginate, and other emerging PSCs. The review will detail their rational design strategies and highlight their applications in enhancing imaging modalities for improved GBM diagnosis, augmenting the efficacy of diverse therapeutic strategies (including chemotherapy, gene and RNA therapy, and combination therapies), and developing integrated theranostic systems. By focusing on these aspects, this review seeks to underscore the promise of PSC-based nanomedicines in shaping the next generation of management strategies for GBM.

## 2. Overcoming the BBB for GBM Therapy: Nanocarrier Strategies and the Role of PSCs

A major obstacle in GBM therapy is the efficient delivery of anticancer agents across the BBB. The tight junctions characteristic of the BBB, formed by endothelial cells, pericytes, and astrocytes, significantly hamper the entry of many therapeutic compounds, particularly large or hydrophilic chemotherapeutics, into the brain parenchyma 7, 8. This barrier meticulously regulates the passage of substances, protecting the central nervous system (CNS) but concurrently limiting the efficacy of systemic treatments for brain malignancies. Nanocarrier-based systems represent a promising avenue to surmount this biological obstruction, aiming to improve drug concentrations at the tumor site while minimizing systemic toxicity.

Nanocarriers can employ several mechanisms to facilitate their passage across the BBB. In instances where the BBB integrity is compromised, nanocarriers, typically ranging from 1 to 100 nm in size (many clinically relevant NPs are larger than 100 nm), may utilize passive diffusion to enter the brain tissue. This is often the case for regions of disrupted vasculature that are associated with the GBM microenvironment changes (a phenomenon sometimes related to the enhanced permeability and retention, or EPR effect). However, the heterogeneity of BBB disruption in GBM necessitates more sophisticated approaches for consistent and widespread delivery.

For areas where the BBB remains more intact, active transport mechanisms are crucial. These include adsorptive-mediated transcytosis (AMT), which is often triggered by electrostatic interactions between cationic nanocarriers and the negatively charged glycocalyx and phospholipid head groups on the luminal surface of brain capillary endothelial cells. Carrier-mediated transport (CMT) offers another route, utilizing endogenous transporters designed for essential nutrients like glucose, amino acids, and nucleosides, although this pathway is generally more suited for smaller molecules or drugs that structurally mimic these native substrates. A more widely exploited active mechanism for nanocarriers is receptor-mediated transcytosis (RMT). This highly specific pathway involves the binding of ligands, purposefully conjugated to the nanocarrier surface, to receptors abundantly expressed on BBB endothelial cells. Commonly targeted receptors include those for transferrin (TfR), low-density lipoprotein (LDL) receptor-related protein 1 (LRP1), lactoferrin (LfR), and insulin receptors 9. Upon ligand binding, the nanocarrier is internalized via endocytosis, transported across the endothelial cell, and subsequently exocytosed into the brain interstitium.

Beyond these direct transport mechanisms, stimuli-responsive nanocarriers present another sophisticated strategy. These systems are engineered to remain relatively inert and stable in systemic circulation, minimizing premature drug release or off-target interactions. However, these nanocarriers undergo specific changes upon encountering specific triggers within the brain microenvironment. Triggers include the acidic pH characteristic of tumor tissues (around pH 6.5 or lower), altered redox gradients (e.g., elevated glutathione levels), or the presence of specific enzymes like matrix metalloproteinases. Externally applied stimuli, such as ultrasound or magnetic fields, can also induce these changes. These changes can include drug release, conformational alteration leading to enhanced cell uptake, or degradation of the carrier matrix. This targeted response thereby enhances the spatiotemporal control of drug delivery across the BBB or within the brain tissue itself, aiming to maximize therapeutic efficacy while reducing systemic side effects 3.

Among the materials explored for traversing the BBB, PSCs (e.g., chitosan, HA, dextran, alginate) offer distinct advantages due to their inherent biocompatibility, biodegradability, and versatile chemical modifiability 2, 10. Crucially, specific PSCs leverage distinct transport mechanisms to overcome the BBB and BBTB. Chitosan, a cationic polymer, utilizes AMT; its positive charge facilitates electrostatic interactions with the negatively charged endothelial luminal surface, promoting transcellular passage 3. Conversely, Hyaluronic Acid (HA) engages RMT by specifically binding to CD44 receptors, which are overexpressed on both activated brain endothelial cells and GBM cells, thereby enabling dual BBB-traversal and tumor targeting. Other PSCs, such as dextran, serve as excellent stealth coatings to prolong circulation or can be functionalized with external ligands (e.g., transferrin, angiopep-2) to induce RMT 9. By tailoring these physicochemical properties—specifically size (optimally 30-150 nm), surface charge, and ligand density—PSC nanocarriers can be engineered to maximize brain accumulation while minimizing systemic clearance 11, 12.

The successful transit of PSC nanomedicines across the BBB and their subsequent therapeutic action are critically dependent on the meticulous control and optimization of their physicochemical properties and functional attributes. By tailoring nanocarrier size, surface charge, ligand attachments, and responsiveness to stimuli, researchers aim to improve BBB transport, enhance drug accumulation in GBM tissue, and control drug release kinetics, thereby maximizing therapeutic efficacy while minimizing adverse effects 4, 11. Precise control of nanoparticle size is vital for effective BBB penetration and favorable pharmacokinetics. While smaller PSC-based NPs (e.g., <100 nm) may more readily diffuse through disrupted tight junctions or fenestrations in leaky BBB regions and are generally favored for endocytic uptake, extremely small constructs (<10 nm) can suffer from rapid renal clearance, leading to a significantly reduced circulation time and insufficient opportunity for BBB interaction 12, 13. Conversely, very large NPs (>200-300 nm) may be rapidly cleared by the RES or may not efficiently cross the BBB even via active transport. Therefore, many PSC nanocarriers are designed with a diameter in the range of 30-150 nm to strike an optimal balance between prolonged systemic residence, avoidance of rapid clearance, and effective brain diffusion or transport.

The surface charge of PSC nanocarriers also significantly influences their interaction with the BBB and systemic fate. Moderately cationic chitosan-based carriers, for example, can leverage their positive charge to encourage AMT. However, an excessively high positive charge might trigger significant nonspecific binding to plasma proteins and blood cells, leading to aggregation, opsonization, and rapid immune clearance, or even endothelial toxicity. Strategies to modulate surface properties and mitigate these issues include coating with neutral, hydrophilic polymers like polyethylene glycol (PEG), known as PEGylation, which creates a steric barrier reducing protein adsorption and prolonging circulation. Partial grafting with other PSCs like HA can also serve a similar purpose or introduce specific targeting functionalities.

The attachment of specific ligands to the surface of PSC nanocarriers is a cornerstone strategy to promote active transport across the BBB via RMT and to enhance tumor cell recognition. Commonly employed ligands include transferrin (Tf), which binds to the highly expressed TfR on brain endothelial cells; angiopep-2, a peptide that targets LRP1; and various antibodies or antibody fragments directed against BBB-specific receptors. For instance, modifications of chitosan or HA with transferrin have been shown to improve NP passage across *in vitro* and *in vivo* BBB models. Cell-penetrating peptides (CPPs), such as TAT peptide or penetratin, can also be conjugated to PSC matrices to encourage deeper tissue uptake and accumulation in glioma sites, possibly by transiently modulating tight junction integrity or by promoting direct membrane translocation or enhanced endocytosis. Multiple reports indicate that such peptide-functionalized carriers exhibit greater fluorescence detection in the brain or more potent anticancer responses in glioma models compared to their unmodified counterparts, emphasizing the therapeutic advantage of active ligand conjugation 14, 15.

Incorporating stimuli-responsive elements into PSC nanocarriers allows for controlled drug release or activation specifically at the tumor site, triggered by the unique tumor microenvironment or by external interventions. For pH-responsive platforms, linkages that are stable at physiological pH (7.4) but hydrolyze or alter conformation in the acidic conditions of GBM tissues (pH ~6.5 or lower intracellularly) are employed. This prevents premature drug leakage during circulation and ensures site-specific activation. For example, mesoporous silica NPs coated with PSC layers have demonstrated significantly higher drug release under acidic conditions compared to neutral pH. Magnetic/pH-sensitive graphene oxide-chitosan microspheres loaded with temozolomide (TMZ) showed nearly doubled drug release at pH 4.5 relative to neutral conditions, a release further amplified by a moderate external magnetic field 16. Redox-sensitive PSC materials leverage the significantly elevated GSH levels found in tumor cells compared to the extracellular environment. These carriers often incorporate disulfide linkages (-S-S-) within their structure or as crosslinkers. These bonds remain stable in the low-GSH environment of the bloodstream but are rapidly cleaved in the high-GSH reductive intracellular milieu of cancer cells, leading to carrier disassembly and burst-like drug release 17. For instance, disulfide-crosslinked chitosan or dextran NPs can be designed to selectively deliver chemotherapeutic agents upon entering tumor cells. One study utilized a porphyrin-based metal-organic framework (MOF) crosslinked with HA that responded to high GSH levels in GBM tissue, releasing porphyrin-MOF NPs capable of generating reactive oxygen species (ROS) under ultrasound irradiation for sonodynamic therapy, and co-delivering L-cysteine to enhance tumor cell apoptosis. However, variations in redox gradients among individual patients and different tumor regions remain a challenge for achieving reproducible therapeutic outcomes with these systems. Furthermore, a critical limitation observed across chitosan studies is the trade-off between BBB penetration and toxicity. While increasing the degree of deacetylation and positive charge enhances absorptive-mediated transcytosis, it concurrently increases the formation of a protein corona in the bloodstream. This leads to rapid clearance by the reticuloendothelial system (RES) and potential neurotoxicity, a contradiction that explains why high-charge formulations often succeed *in vitro* but fail to maintain therapeutic concentrations in orthotopic animal models.

Exogenous triggers such as ultrasound (US) and magnetic fields further broaden the therapeutic potential. Focused ultrasound, often in conjunction with microbubbles, can transiently and locally disrupt the BBB, improving NP penetration. US can also stimulate faster drug release from carriers sensitive to mechanical or thermal energy. For instance, PSC-based silica carriers loaded with doxorubicin (DXR) displayed accelerated release upon ultrasonic stimulation, contributing to higher intratumoral accumulation and reduced cardiotoxicity. Magnetic fields can guide PSC-coated magnetic NPs (e.g., iron oxide cores) to the tumor site or trigger drug release. In magnetothermal therapy, these magnetic elements generate localized heat under an alternating magnetic field (AMF), directly damaging glioma cells (typically at 42-45°C) and enabling temperature-dependent drug release from thermosensitive PSC carriers. This hyperthermia can also increase tumor permeability and sensitize cells to chemotherapy or radiotherapy. Iron-containing PSC-NPs can also participate in chemodynamic therapy via the Fenton reaction, where released iron ions catalyze the conversion of endogenous H₂O₂ into highly toxic hydroxyl radicals, specifically within the acidic tumor microenvironment.

Beyond systemic administration, alternative delivery routes are being explored for PSC nanomedicines. Intranasal delivery, for example, exploits the direct nose-to-brain pathways (olfactory and trigeminal nerves) to bypass the BBB to some extent. Chitosan's mucoadhesive properties are particularly beneficial here, prolonging residence time in the nasal cavity and enhancing absorption. Chitosan-decorated poly(lactic-co-glycolic acid) (PLGA) NPs loaded with carmustine (BCNU) nearly tripled brain drug levels in rats via intranasal administration compared to intravenous injection 18. Similarly, β-cyclodextrin-chitosan coatings on gold-iron oxide NPs demonstrated efficient intranasal uptake and led to marked survival benefits in mouse glioma models 19. Localized delivery using PSC-based hydrogels implanted into post-resection cavities is another promising strategy. These hydrogels can act as depots for sustained, localized release of chemotherapeutics or immunomodulators, minimizing systemic toxicity and targeting residual tumor cells. For instance, a semi-synthetic PSC hydrogel loaded with ruxolitinib provided sustained release for 14 days and showed strong *in vitro* GBM cell-growth inhibition 20. Cellulose nanocrystal hydrogels delivering paclitaxel (PTX) have also demonstrated measurable tumor suppression 21.

GBM is characterized by pronounced inter- and intratumoral heterogeneity, which includes variations in BBB integrity, receptor expression profiles, and microenvironmental conditions across different tumor regions and among patients 22. Effective therapeutic strategies must therefore be adaptable and capable of addressing drug delivery to both intact and partially leaky BBB regions, as well as targeting diverse tumor cell populations. To enhance specificity and efficacy, dual-targeting approaches are being developed. These equip PSC nanocarriers with one ligand for BBB passage (e.g., transferrin or angiopep-2) and another for binding to receptors overexpressed primarily on tumor cells (e.g., integrin-binding RGD peptides for neoangiature or glioma cells, or CD44-binding HA for glioma cells). This strategy aims to improve drug accumulation and retention specifically within invasive glioma cells, thereby enhancing overall therapeutic efficacy 23, 24. For example, HA-based nanomicelles modified with angiopep-2 and incorporating a hypoxia-sensitive moiety for triggered release have been developed. One such system, also utilizing Tween 80 for enhanced BBB passage, achieved a 7.6-fold increased transport across an *in vitro* BBB model compared to formulations lacking Tween 80 25.

PSC nanocarriers are also being engineered as theranostic platforms, integrating imaging and therapeutic functions into a single complex. Dextran-coated iron oxide NPs delivering antisense oligonucleotides permit concurrent MRI tracking and gene silencing in GBM 26. Multifunctional chitosan-coated magnetite graphene oxide systems, grafted with gastrin-releasing peptide ligands and loaded with doxorubicin, demonstrated decreased tumor burden under an external magnetic field, showcasing combined targeting, imaging capability, and therapy 27.

PSC nanocarriers are advancing gene and RNA therapy for GBM by securely encapsulating and transporting nucleic acids such as siRNA, miRNA, or plasmid DNA. The inherent biocompatibility of PSCs like chitosan and HA typically reduces immunogenic concerns associated with viral vectors. Surface modifications, including PEGylation or the addition of targeting ligands like transferrin, further extend circulation time, curb nonspecific adsorption, and improve selectivity for glioma cells 28, 29.

Furthermore, some PSCs (e.g., certain β-glucans, chitosan) possess intrinsic immunostimulatory properties, which can be leveraged to augment the effects of co-delivered immunotherapeutic agents like checkpoint inhibitors, cytokines, or tumor-associated antigens. Such carriers can accumulate in GBM tissue, help activate local anti-tumor immune responses by engaging innate immune cells, and synergize with conventional chemotherapy or radiotherapy. Hybrid constructs incorporating immunomodulators like CpG oligodeoxynucleotides within PSC matrices are also being explored 30.

Ensuring the safety of these nanoplatforms is paramount for clinical translation. This necessitates thorough preclinical evaluation, including comprehensive acute and chronic toxicity studies, immunogenicity assessments, and detailed biodistribution profiles. Noninvasive imaging techniques, such as magnetic resonance imaging (MRI) and near-infrared (NIR) fluorescence imaging, are invaluable tools for monitoring carrier localization, BBB crossing efficiency, tumor accumulation, and overall safety profiles *in vivo*.

As research in this domain continues to advance, PSC-based nanomedicines hold significant promise for transforming GBM management. They offer a versatile platform that can be engineered for systemic or local administration, employed as standalone delivery systems, or used in combination with other therapeutic modalities such as immunotherapies, focused ultrasound, or radiotherapy 5. Achieving deeper penetration into the brain parenchyma, beyond the often-compromised tumor core and into invasive tumor margins, requires a holistic approach. This involves capitalizing on passive diffusion where possible, but more critically, harnessing receptor-mediated uptake mechanisms and exploiting environmental triggers or external stimuli to concentrate anticancer agents within GBM cells while minimizing off-target toxicity. The systematic assessment and optimization of all relevant design factors—including PSC type and molecular weight, nanoparticle size and morphology, surface charge and hydrophilicity, ligand choice and density, drug loading capacity, and release kinetics—are crucial for maximizing BBB penetration and subsequent tumor uptake and therapeutic effect 10. Continued *in vivo* experiments in increasingly sophisticated and clinically relevant orthotopic GBM models are essential to elucidate how these multifaceted parameters collectively influence therapeutic efficacy and to validate safety. Ongoing efforts will likely focus on refining ligand specificity, developing multi-stimuli responsive systems for even greater spatiotemporal control, integrating combination therapies (e.g., chemo-immunotherapy or chemo-gene therapy), and establishing standardized, reproducible manufacturing and characterization protocols. By meticulously refining each aspect of PSC-driven delivery and rigorously validating their performance, these nanocarriers are poised to remain at the forefront of innovative strategies for crossing the BBB and offering safer, more effective treatments for the formidable challenge of GBM.

## 3. PSC-Based Strategies for GBM Imaging

PSC-based carriers are increasingly explored for GBM diagnostics, effective imaging. However, is fundamentally limited by the BBB, a highly selective interface that restricts the passage of most agents from systemic circulation into the brain parenchyma 31-33. Consequently, significant research focuses on engineering nanocarriers, particularly PSCs, to leverage biological transport mechanisms. These biocompatible and structurally versatile macromolecules can be functionalized to cross the BBB and are readily conjugated with imaging agents—such as fluorescent dyes, gadolinium (Gd)-based contrast agents, or NPs—to enable sensitive, noninvasive tracking and improve tumor boundary delineation 34-36.

As detailed in Section 2, PSC nanoplatforms traverse the BBB primarily by leveraging RMT (targeting receptors like TfR1, LRP-1, and GLUT1) or AMT strategies 37-42. Biomimetic strategies, including hyaluronic acid (HA) coatings to target CD44 on GBM cells 43 or cell-membrane cloaking (e.g., RBCs, EVs) to evade immune detection, also enhance delivery 44. Additionally, physical methods, notably focused ultrasound (FUS) with microbubbles, can transiently disrupt the BBB to significantly boost nanocarrier accumulation 45.

Once across the BBB, these PSC platforms deliver diagnostic payloads for various imaging modalities, dominated by Magnetic Resonance Imaging (MRI) and optical imaging (OI) due to their clinical maturity and safety (Table 1) 46. MRI remains the clinical gold standard, providing high-contrast anatomical information. PSCs incorporating superparamagnetic iron oxide NPs (IONPs) or Gd-based agents enable enhanced *T*_1_-weighted or *T*_2_-weighted imaging, allowing for precise tracking of nanoparticle accumulation and longitudinal monitoring of treatment response 47, 48. Complementarily, OI, particularly Near-Infrared (NIR) fluorescence, is vital for real-time intraoperative guidance, offering the high spatial resolution needed to accurately delineate tumor margins and ensure maximal safe surgical resection 49, 50.

Emerging modalities are increasingly integrated into PSC designs to provide functional and quantitative data. Positron Emission Tomography (PET) and Single-Photon Emission Computed Tomography (SPECT) offer exceptional sensitivity and unlimited tissue penetration, overcoming the depth limitations of OI 51. By stabilizing radiotracers, PSCs can map molecular processes, such as amino acid uptake [^18^F]-FET PET), to assess tumor metabolism undetected by structural MRI 52, 53. Hybrid PET/MRI systems combine the anatomical resolution of MRI with the quantitative sensitivity of PET 54. Furthermore, acoustic modalities offer unique capabilities. Photoacoustic imaging (PAI) leverages PSCs carrying strong light absorbers (e.g., gold NPs) to generate high-resolution, non-invasive images 55. Ultrasound (US) serves a dual role: beyond its use in FUS-mediated BBB disruption, it can be used for imaging or as an external trigger for controlled drug release from engineered PSC nanobubbles 56, 57.

In summary, while MRI and OI remain the cornerstones of clinical translation for PSC-based imaging, the field is advancing toward sophisticated multimodal systems. These platforms integrate the quantitative metabolic insights of PET/SPECT and the functional capabilities of photoacoustic and ultrasound imaging 58, 59. The modular nature of PSCs allows for the incorporation of BBB-targeting ligands, multiple imaging moieties, and stimuli-responsive features (e.g., pH/redox sensitivity) to boost signal strength specifically within the tumor. This integrated approach aims to overcome the limitations of single-modality imaging, enabling more precise diagnosis, real-time intervention, and rigorous monitoring of therapeutic outcomes in GBM 60, 61.

### 3.1 PSC-Based Probes for Magnetic Resonance Imaging

Multiple studies demonstrate that HA-conjugated IONPs exhibit superior *T*_2_-weighted contrast compared to non-targeted controls, directly correlated to their accumulation in CD44-overexpressing GBM cells 62. Another investigation used HA conjugation to low-molecular-weight SPIONs, demonstrating strong *T*_2_^*^ signal changes that correlated with significant NP accumulation in GBM cells but minimal uptake in healthy fibroblasts 65. Shared characterization methods typically include DLS, zeta potential, and various spectroscopic analyses, while *in vitro* experiments commonly employ U87 glioma cells to confirm preferential internalization 63, 64.

**Figure 2:**
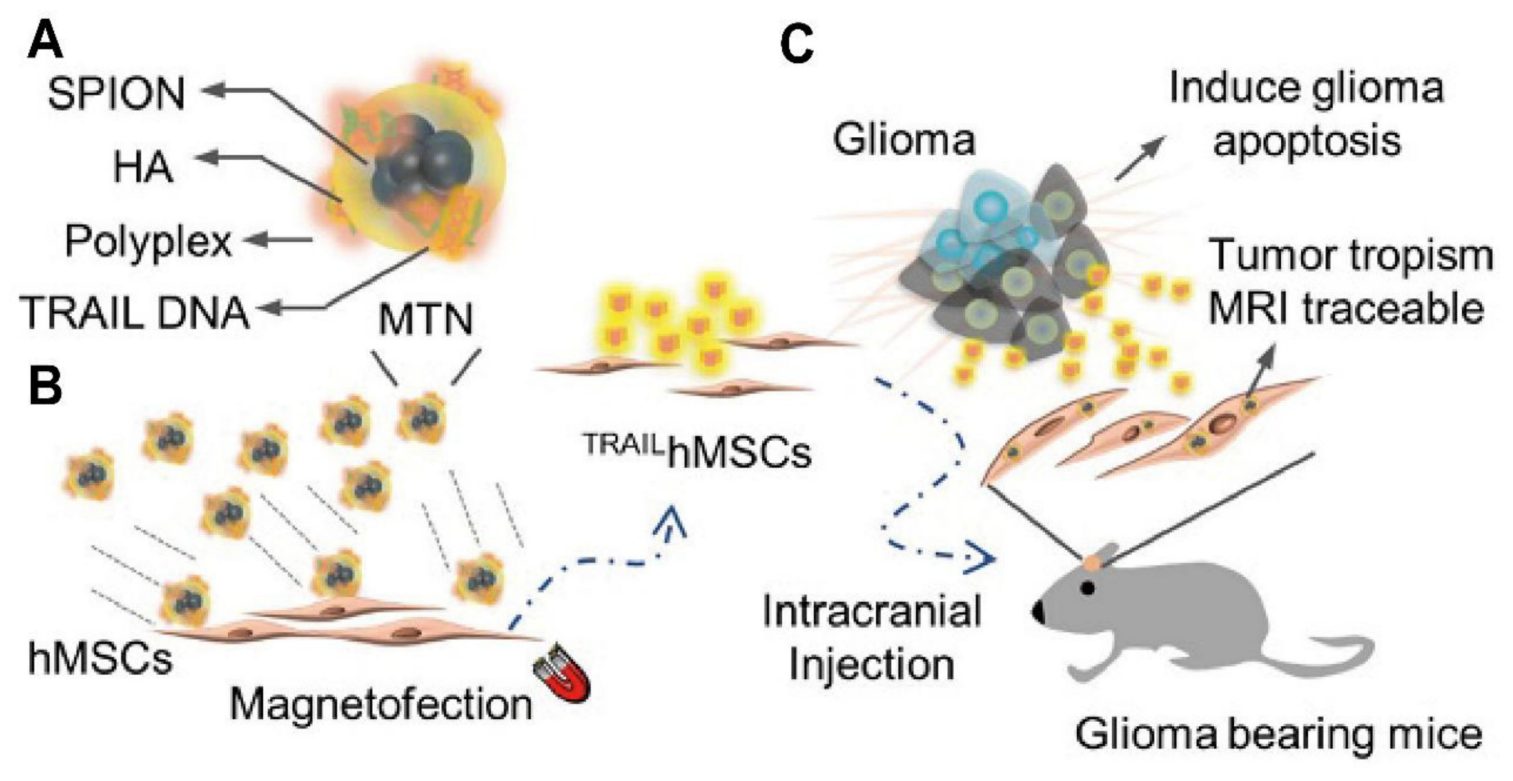
Schematic illustration of the generation and therapeutic application of TRAIL-engineered human mesenchymal stem cells (TRAILhMSCs) for glioma therapy.** (A)** hMSCs are modified via magnetofection. This process utilizes a magnetic nanoparticle complex consisting of SPIONs, HA, polyplex and plasmid DNA encoding for TRAIL, along with a magnetofection agent (MTN). **(B)** The resulting TRAILhMSCs are engineered for enhanced tumor tropism and MRI traceability. **(C)** Following intracranial injection into glioma-bearing mice, TRAILhMSCs migrate towards glioma. The expressed TRAIL protein on the surface of the hMSCs induces apoptosis in glioma cells, leading to tumor regression, while the SPIONs allow for non-invasive tracking of the cells via MRI. Adapted with permission from reference 63. Copyright 2019 Ivyspring International Publisher (open access).

Several HA-coated IONP systems feature similar size distributions, generally ranging from 10 to 100 nm, and exhibit low cytotoxicity *in vitro*. Dual-targeting ligands, such as folic acid, can further enhance receptor-mediated binding to specific GBM subpopulations, although other designs leverage HA alone or in combination with surface peptides to improve BBB crossing or intratumoral penetration 65. Despite various promising *in vitro* results, most studies rely on small animal models that may not capture the complexity of human gliomas, highlighting the importance of larger-scale investigations. Exploiting the CD44-mediated uptake and magnetically responsive cores of these HA-based iron oxide probes shows promise for precise tumor visualization, targeted drug delivery, and potentially broader clinical applications. Crucially, a distinction must be made here: while many HA-iron oxide formulations serve solely as passive *T*_2_-weighted contrast agents, true theranostic designs further capitalize on the magnetic core for hyperthermia or conjugate chemotherapeutics (e.g., Doxorubicin) to the PSC shell. This transition from a diagnostic probe to a theranostic agent enables the simultaneous monitoring of tumor boundary reduction and therapeutic response. Future work might delineate strategies for integrating multiple targeting moieties or therapeutic payloads to amplify their imaging and cytotoxic effects, but efforts must address safety and pharmacokinetics in clinically relevant settings (Figure 2A-C) 62, 63.

Chitosan and dextran have also been widely adopted to stabilize, functionalize, or crosslink iron oxide cores for MRI-guided GBM interventions. Dextran coatings often confer colloidal stability, reduce cytotoxicity, and allow traceability under MRI. For example, crosslinked dextran-coated IONPs loaded with antisense oligonucleotides enabled image-guided tracking and showed therapeutic potential in orthotopic glioma models. Another study assessed Chitosan-dextran superparamagnetic NPs in rodent glioma models, reporting a uniform particle size of about 55 nm and significant *T*_2_ contrast enhancement in the tumor region 64. These findings indicate that Chitosan can significantly improve cellular uptake and tumor retention, although its elevated charge requires optimization to mitigate potential off-target effects.

Other works have examined chitosan alone as a coating agent for Fe_3_O_4_, emphasizing its potential in hyperthermia and its role as a dual-action chemotherapeutic carrier 65, 66. Comparisons reveal that dextran-stabilized systems frequently exhibit higher stealth characteristics in blood-based assays, whereas chitosan formulations demonstrate stronger cellular internalization. Nonetheless, many investigations use limited *in vitro* experiments or small-scale animal studies, so the generalizability of robust internalization or hyperthermic outcomes to complex human tumors remains uncertain. Furthermore, specialized clinical equipment may be required for approaches involving strong external magnetic fields or hyperthermic conditions, underscoring the need for continued research on scalability and safety.

Other PSCs including cyclodextrins and CMC, have likewise demonstrated potential to enhance metal-based probes for GBM theranostics. One example employed gold-iron oxide nanohybrids coated with β-cyclodextrin-chitosan polymers for siRNA or miRNA loading, enabling combined optical and magnetic imaging, along with gene-silencing properties. This platform supported fluorescence and MRI-based molecular imaging and showed the capacity to bypass physiological barriers via intranasal delivery. Another approach used cyclodextrin-bearing IONPs conjugated to CTX, reporting improved efficacy against drug-resistant GBM cells and stable dispersion under physiological conditions 67. These findings highlight cyclodextrins' usefulness in entrapping hydrophobic payloads or enabling host-guest interactions, potentially broadening the range of anticancer agents that can reach the brain.

CMC has been used to cap and functionalize QDs or to dope iron oxide NPs with chemotherapeutic agents 68. Attaching CMC to ZnS or AgInS_2_ cores yields nanocrystals with a stable, water-dispersed shell and robust fluorescence, allowing cell labeling and monitoring in glioma models. Similar systems that conjugate DXR to carboxymethylated PSC shells exhibit pH-sensitive drug release, promoting payload release in the acidic tumor environment 69. Though promising, concerns remain regarding toxic byproducts released during quantum dot or cyclodextrin-based NP degradation, warranting rigorous *in vivo* toxicity assessments. Ensuring safe accumulation patterns and reliable therapeutic outcomes in more advanced models remains critical before clinical translation.

Less common but still compelling PSCs such as tragacanth and PST001 have also been investigated for metal NP stabilization in GBM applications. One study employed tragacanth to synthesize NiO nanosheets (18-43 nm), which displayed moderate photocatalytic dye degradation (60-82%) and cytotoxic effects against U87MG cells at higher concentrations, with an IC50 of 125 µg/mL 70. These nanosheets exhibited favorable crystallinity and surface properties, suggesting potential for concurrent imaging based on NiO's optical characteristics, though direct MRI evidence remains limited. Another group developed DXR-loaded carboxymethylated PST001-coated iron oxide NPs that showed improved biocompatibility, enhanced intracellular uptake, and significant reactive oxygen species generation in both two-dimensional and 3D culture models 71. While these materials demonstrate innovative surface chemistries and promise for GBM therapy, their ability to cross the BBB or deliver clinically meaningful theranostic performance requires further large-scale *in vivo* testing 72.

Collectively, enlisting these diverse PSCs to coat iron oxide or other metal NPs has enabled significant advancements in imaging contrast, tumor selectivity, and drug delivery. HA-MSA2 systems stand out for their CD44-mediated uptake and strong MRI signals, while chitosan offers higher uptake efficiency through electrostatic interactions, and dextran coatings generally improve circulation time. CD and CMC-based platforms broaden the scope of loaded therapeutics and imaging modalities, although attention to potential toxic byproducts remains paramount. Less common PSCs contribute distinctive surface chemistries but still need substantial *in vivo* validation to confirm their translational potential. Ongoing efforts must address biodistribution, immunogenicity, and scalability, ensuring that these PSC-based nanosystems can offer safe and effective theranostic solutions for GBM in clinical practice 73.

### 3.2 PSC-Based Probes for Optical (Fluorescence and Near-Infrared) Imaging

BAA-HA-MSA2-MSA2 remains a promising targeting ligand for GBM therapy, particularly when combined with NIR imaging for enhanced tumor visualization. A multifunctional nanocomplex exemplifying this role integrates B NSs and Au NPs with Ag2S, then caps the construct with BAA-BAA-HA-MSA2-MSA2 74. The system leverages the NIR-II fluorescence from Ag2S to enable deep-tumor imaging, while photothermal and sonodynamic activities bolster tumor ablation. The HA coating facilitates tumor-specific accumulation, and US-assisted microbubble disruption transiently opens the BBB, improving localization in GBM tissue. Although researchers observed robust anti-tumor immune responses and immunogenic cell death, the limited number of *in vivo* subjects constrains broader clinical extrapolation. Nonetheless, integrating HA with NIR-based approaches offers an encouraging direction for tumor-targeted imaging and therapy with greater spatiotemporal precision.

CMC is a versatile PSC matrix that can incorporate QDs for fluorescence-based imaging in GBM cells. For instance, cationic ε-poly-l-lysine-based ZnS QDs assemble with anionic CMC-encapsulated magnetite nanozymes via electrostatic interactions, forming hybrid organic-inorganic stimuli-responsive nanoplexes 75. Unlike the purely diagnostic QDs discussed previously, these nanoplexes represent a dual-function theranostic platform: the QDs provide bright luminescence for tracking, while the magnetite nanozymes enable concurrent chemodynamic therapy. The QDs provide bright luminescence for imaging in both two-dimensional and 3D spheroid models, while the magnetite nanozymes enable chemodynamic therapy and magnetohyperthermia. Another approach involves supramolecular QD-biopolymer-drug assemblies, where ZnS QDs capped with CMC are conjugated with DXR, facilitating simultaneous bioimaging and chemotherapy. A parallel strategy uses CMC functionalized with cell-penetrating and pro-apoptotic peptides to complex fluorescent AIS semiconductor nanocrystals, resulting in multimodal imaging and potent cytotoxic effects against GBM cells 76. Although these studies converge on the capacity of CMC to stabilize QDs, NP size and luminescence intensity vary due to differences in QD core composition and the inclusion of therapeutic moieties. A key limitation arises from predominantly *in vitro* protocols, which may not capture *in vivo* pharmacokinetics or fully represent the tumor microenvironment. Nonetheless, these findings collectively highlight the potential of CMC-based fluorescent nanoplatforms to advance theranostic applications in GBM.

Dextran, a commonly used PSC for NP coating, enhances the stability, biocompatibility, and imaging capabilities of IONPs. One dextran-based nanosystem featured crosslinked iron oxide cores labeled with Cy5.5 for dual-modal MRI and optical imaging. Its 25 nm cores and crosslinked shell allowed preferential accumulation in orthotopic GBM following intravenous injection, while concurrently delivering antisense oligonucleotides against oncogenic miRNA. Although variability in dextran thickness and crosslinking density can influence biodistribution and clearance, this platform demonstrated transvascular passage and consistent imaging signals in glioma-bearing models. Optimizing coating strategies or further tailoring the shell could improve targeting accuracy while preserving robust imaging contrast.

Chitosan, another abundant PSC, can be adapted for tumor-targeted drug delivery and fluorescence-based imaging. One example employs selenium-Chitosan NPs loaded with TMZ, coated with Eudragit® RS100 polymer, and tracked through encapsulated curcumin 77. The innate fluorescence of curcumin confirms NP uptake by glioma cells, and Chitosan-based carriers lower the IC50 while downregulating TMZ resistance genes. However, curcumin's photobleaching limits extended imaging capability. Still, combining Chitosan-mediated tumor targeting with fluorescent labeling offers a promising method for monitoring drug release and therapeutic efficacy in GBM 78.

Collectively, PSC-based vehicles composed of HA, CMC, dextran, or chitosan present complementary strategies for coupling robust imaging with tumor-directed therapies in GBM. By incorporating fluorescent or NIR markers, each system enables real-time tumor visualization, more accurate drug delivery, and enhanced assessment of therapeutic effectiveness 79. Although preclinical studies underscore their promise, variations in particle composition and the complexity of the BBB remain key challenges. Standardizing imaging parameters, addressing scale-up, and evaluating long-term safety are essential next steps in translating these platforms to clinical settings. Continued research may ultimately yield safe, effective, and clinically feasible PSC-based nanotechnologies for targeted GBM therapy.

## 4. PSC-Based Systems for GBM Therapy

PSCs are increasingly employed to deliver chemotherapeutic drugs and genetic materials to GBM cells, owing to their biocompatibility, biodegradability, and adaptable chemical structures. These properties support the development of nanocarriers that address critical GBM challenges, including the BBB, tumor heterogeneity, and drug resistance. By modulating parameters such as molecular weight, substitution degree, and surface properties, PSC nanocarriers achieve enhanced stability, targeted delivery, and prolonged circulation, thereby improving the therapeutic index. Their utility spans a wide range of payloads, from conventional anticancer drugs to gene-based treatments, all while mitigating off-target effects. Furthermore, conjugations with PEG or targeting ligands augment immune evasion and strengthen site-specific accumulation. Recent studies have explored various PSC platforms, capitalizing on stimuli-responsive linkers to enable controlled release within the tumor microenvironment. Through these design strategies, PSC-based platforms show promise in addressing GBM's complex pathology and improving patient outcomes.

### 4.1 Chitosan-based approaches

**Figure 3:**
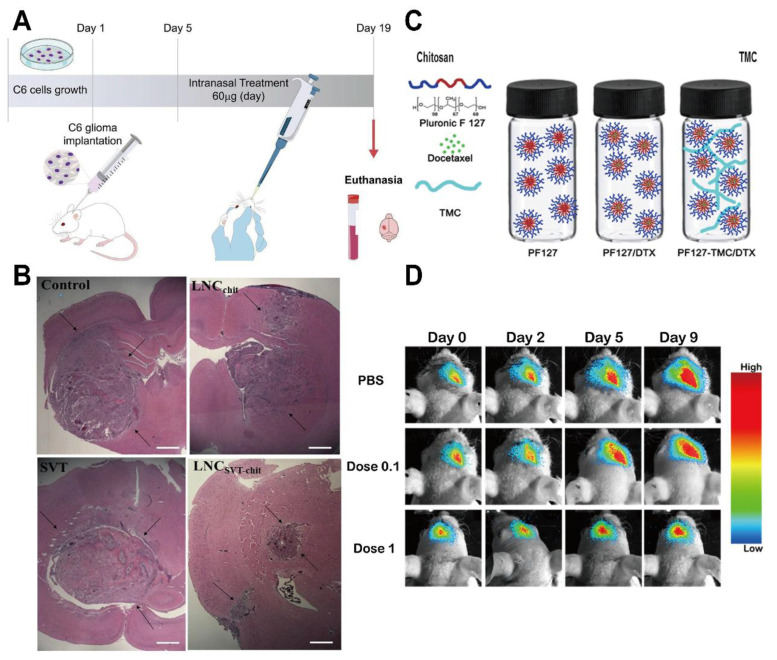
Study of Chitosan in the treatment of glioma. **(A)** Nanocapsules coated with Chitosan and loaded with simvastatin are administered intranasally to avoid the BBB and improve drug delivery efficiency. **(B)** After 14 days of treatment, gliomas in the experimental group were significantly smaller than those in the control group. **(C)** The thermosensitive hydrogel was prepared with PF127 and N,N, N-TMC as raw materials and loaded with DTX for GBM. **(D)** PF127-TMC/DTX gel with high concentration can significantly inhibit the growth of glioma cells *in vitro*. Adapted with permission from reference 81, 82. Copyright 2022 Elsevier B.V. and 2018 Elsevier Ltd., respectively.

**Figure 4:**
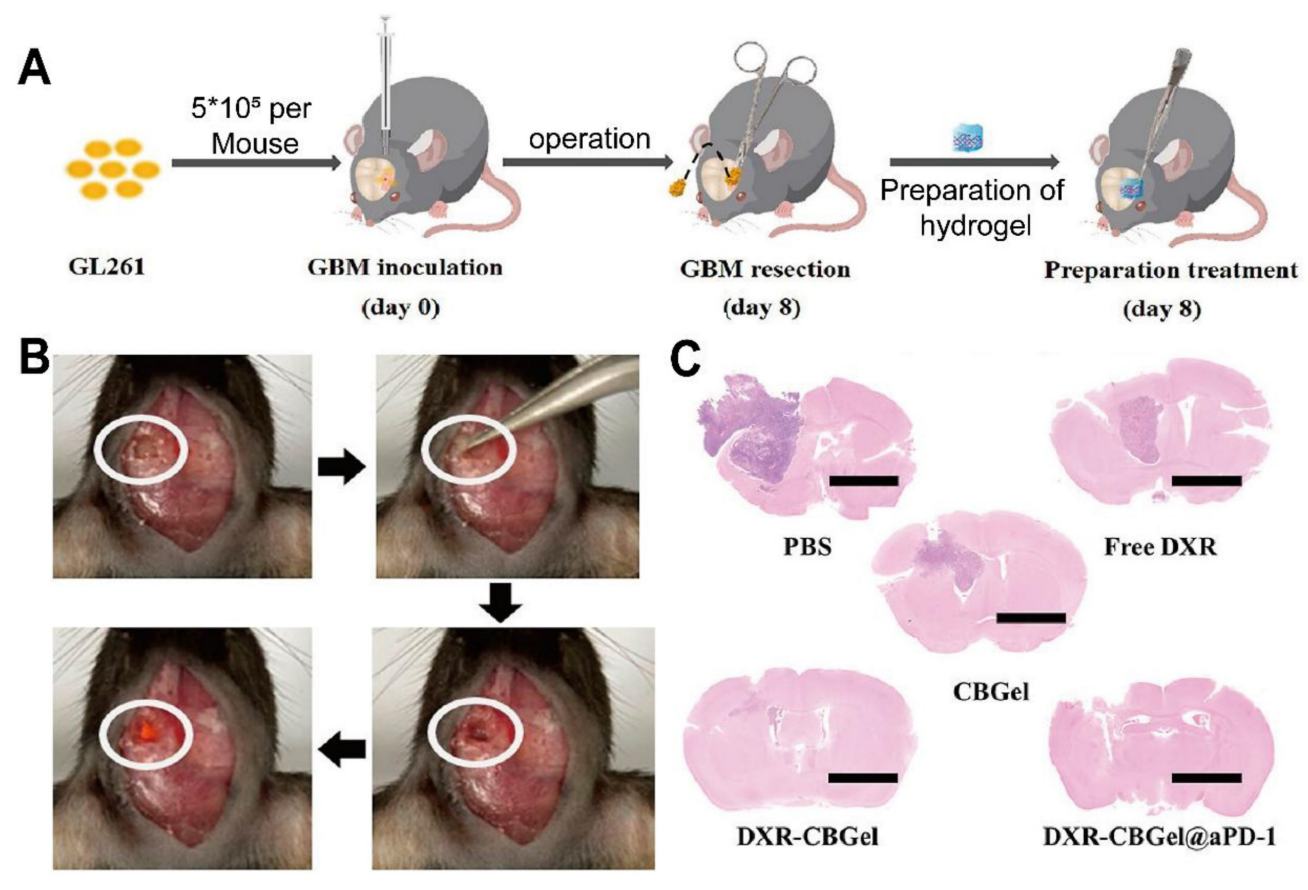
Intracavity-filled Chitosan system significantly suppressed tumor recurrence. **(A)** Study of DOX-loaded bovine serum albumin NPs and chitosan self-crosslinking drug delivery system in glioma recurrence after surgery. **(B)** Tumor resection was performed on the 8th day after tumor inoculation, and drug delivery systems of different groups were implanted in the tumor remnant cavity. **(C)** H&E staining on the 18th day of each group showed that the experimental group had significant inhibition on tumor growth after glioma surgery. Adapted with permission from reference 83. Copyright 2023 American Chemical Society.

Chitosan-based nanocarriers have garnered attention for intranasal and systemic delivery (Table 2) 80. For example, nanocapsules utilizing chitosan's mucoadhesive properties significantly reduced glioma size in mouse models (Figure 3A, B) 81. Other approaches involve thermosensitive hydrogels loaded with drugs like docetaxel (DTX) (Figure 3C), which demonstrated significant inhibition of glioma growth in preclinical models (Figure 3D) 82. Additionally, drug delivery systems using chitosan have been developed for post-surgical local treatment to prevent glioma recurrence (Figure 4A). In such studies, the drug delivery system is implanted into the tumor remnant cavity after resection (Figure 4B), showing significant inhibition of subsequent tumor growth in an animal model (Figure 4C) 83. Beyond small molecule agents, chitosan-based systems can also preserve the biological activity of macromolecules. One study demonstrated that trimethyl chitosan (TMC) NPs encapsulating an IGF-1R inhibitor (IGF-Trap) yielded superior intracerebral drug uptake and prolonged survival in mouse glioma models 84.

Additional research underscores the versatility of chitosan-based carriers in accommodating multiple functional elements. Magnetic graphene oxide modified with chitosan and conjugated to gastrin-releasing peptide allowed dual magnetic and receptor-mediated DXR targeting. This system achieved pH-responsive release, significantly lowering the IC50 in GBM cells 27. Another platform combined silicon NP with a chitosan coating, extending circulation time in tumor-bearing mice and favoring intratumoral accumulation through the EPR effect 85. Chitosan-coated iron oxide NP similarly demonstrated reduced cytotoxicity in C6 glioma cells compared to uncoated variants, implying that the polymeric layers can mitigate metal-associated toxicity while bolstering anticancer efficacy 86. Moreover, surface alterations such as chemical modifications to chitosan can improve its solubility at physiological pH. Trimethyl chitosan (TMC), for example, has garnered special interest for its ease of functionalization, enabling ligand attachment that bolsters targeting efficiency and overall therapeutic potential.

Studies have highlighted TMC-based carriers co-functionalized with ligands such as RGD or folate, which anchor selectively to overexpressed receptors in GBM 87. One investigation described a TMC NP platform functionalized with RGD and L-arginine that directed cytotoxic activity primarily toward cancer cells in a preclinical model, sparing healthy tissues and underlining the specificity conferred by these ligands. Another effort demonstrated folic-acid-conjugated chitosan NP, resulted in improved mucoadhesion and cytotoxic activity *in vitro* 88. Additionally, chitosan has been derivatized via N-alkylation to form micelle-like structures, facilitating translocation of macromolecular payloads across the BBB 89.

In parallel, chitosan's utility extends to functionalization strategies that target specific receptors or enable unique trans-barrier transport. Anti-glypican-1 antibody (AT101)-conjugated chitosan nanobubbles selectively accumulated in glypican-1-overexpressing GBM in a mouse model, significantly augmenting tumor binding relative to unmodified counterparts 90. Triphenylphosphonium (TPP⁺)-conjugated chitosan formulations likewise improved mitochondrial targeting in GBM cells, thereby enhancing the cytotoxic effectiveness of TMZ. A consecutive layering of chitosan and HA in nano-emulsions, further functionalized with a CRT peptide, yielded increased uptake across BBB-cell models by exploiting transferrin receptor binding 91. Explorations of sialic-acid-covered chitosan NPs add another dimension by taking advantage of sialic acid's tumor-targeting capabilities and stabilizing effects.

Beyond nano-scale delivery, chitosan-based hydrogels facilitate localized and sustained drug release, particularly beneficial following glioma resection 92. *In situ* gelation triggered by temperature changes or crosslinking can achieve prolonged retention of therapeutic agents at the tumor site. One example involved an injectable chitosan hydrogel loaded with tumoricidal neural stem cells (iNSCs), resulting in long-term survival benefits and increased median survival in animal studies relative to direct stem cell injections 93. Another formulation employed DXR-loaded liposome-like particles (LLP-DOX) in a chitosan/hyaluronic acid/polyethyleneimine (CHI/HA/PEI) matrix, providing gradual drug release and substantially reducing the viability of three-dimensional (3D) GBM spheroids 94. A further approach used a dual-crosslinked macroporous hydrogel composed of sodium alginate and chitosan, which effectively sequestered GBM cells post-resection in an animal model without overwhelming healthy tissues 95. Additionally, an albumin-based hydrogel crosslinked with chitosan extended intratumoral retention of Dox in a mouse model, leading to immunogenic cell death and minimal systemic toxicity 96. Although these matrices require precise tuning of mechanical properties and may rely on suitably sized resection cavities, they offer a promising route to contain residual glioma cells and reduce the risk of recurrence.

Multi-modal or stimuli-responsive chitosan formulations further broaden therapeutic options by merging magnetic, photothermal, or pH-sensitive functionalities. One pH- and magnetically responsive system incorporated iron oxide NPs into a chitosan-graphene oxide matrix, delivering TMZ under external magnetic field stimulation and accelerating drug release within acidic tumor environments 97. This system functions as a true theranostic platform: the iron oxide core enables *T*_2_-weighted MRI tracking of tumor accumulation, while the chitosan shell concurrently manages the stimuli-responsive release of the chemotherapeutic payload. Others employed NIR photothermal conversion using platinum-gold nanorods layered in a chitosan/polycaprolactone scaffold, which yielded extended TMZ release and a high cell-killing rate in GBM cultures under NIR irradiation 98, 99. In another approach, radiofrequency-triggered hyperthermia in chitosan-coated silicon NPs intensified tumor cell death in preclinical tests. Chitosan is also conducive to photodynamic therapy, exemplified by berberine-based chitosan nanosystems that induced apoptosis in T98G cells while sparing healthy astrocytes 100. Successful integration of these stimuli hinges on precise field delivery and minimal off-target damage, yet the adaptive nature of chitosan indicates that such hurdles may be overcome with design refinements and advanced instrumentation.

Combining therapeutic modalities appears increasingly feasible with chitosan carriers, which can integrate several agents or strategies in a single platform. For instance, chitosan NPs loaded with gemcitabine heightened the susceptibility of glioma cells to TMZ, achieving synergistic apoptotic effects 101, 102. Dual photothermal-chemotherapeutic regimens have also been devised. For example, HA-modified chitosan-lipid NPs co-loaded with cisplatin and iron oxide, were guided externally to glioma tissue and activated by NIR light to enhance cytotoxic potency *in vivo* 103. Curcumin-chitosan nanocomplexes further exemplify the potential for epigenetic reprogramming, as they modulated DNA methyltransferase gene expression and displayed robust dose- and time-dependent cytotoxicity in GBM cells 104. While precise attribution of efficacy to each individual component remains challenging, these multi-pronged systems generally yield greater tumoricidal effects than single-agent regimens.

Collectively, these diverse approaches illustrate chitosan's broad applicability to GBM therapy, ranging from NP constructs and hydrogels to complex multi-modal regimens 105. Three-dimensional tumor models employing chitosan-HA scaffolds emphasize the polymer's promise for both drug delivery and high-throughput drug screening, as the scaffold's pore size modulates GBM cell behavior and resistance 106. Simultaneously, stealthy chitosan-capped silicon NPs capable of extended circulation underscore emerging strategies to prolong half-life and enhance tumor accumulation 107. Despite these encouraging findings, many studies rely on limited preclinical models, do not include standard comparators, or assess only short-term outcomes. Moving forward, comprehensive validation in intracranial models, standardized efficacy metrics, and large-scale manufacturing protocols will be vital for translating chitosan-based nanomedicines into effective clinical solutions against GBM.

### 4.2 HA-based approaches

HA has emerged as a promising PSC-based platform for targeting GBM due to its intrinsic tumor-homing capabilities 108. By selectively binding these receptors, HA-based carriers facilitate the uptake of chemotherapeutics within tumor tissues while minimizing off-target effects 109. Moreover, HA's natural biocompatibility, biodegradability, and relatively low immunogenicity support its application in NP formulations for efficient and well-tolerated drug delivery. Preclinical studies show that HA-based nanocarriers accumulate in GBM cells and exploit local microenvironmental conditions to enable targeted release, ultimately improving intracranial drug distribution. Taken together, these advances underscore HA's potential to serve as a versatile vehicle that can cross the BBB and enhance therapeutic specificity (Table 3).

**Figure 5:**
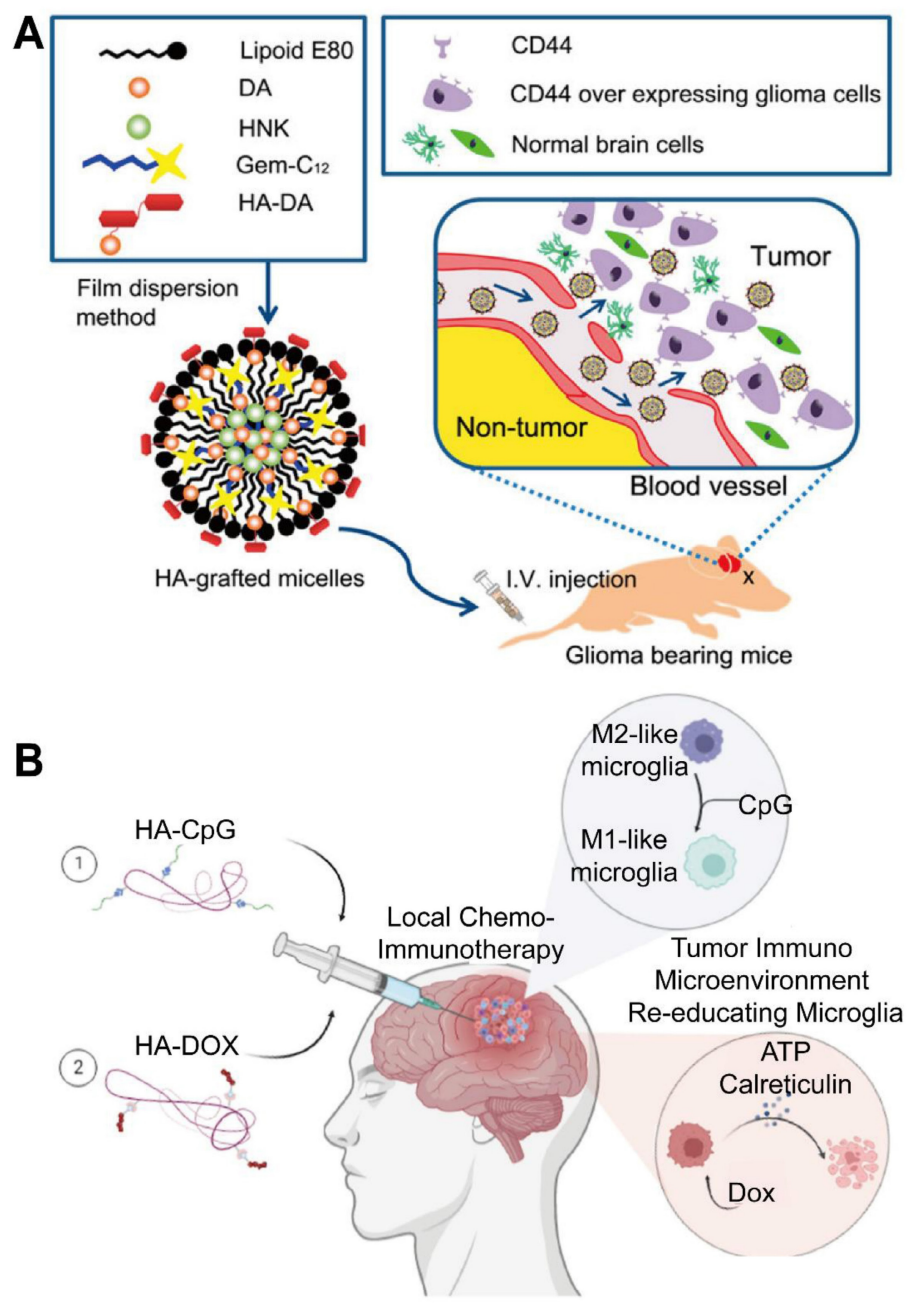
HA-based NPs improve the integration of glioma diagnosis and treatment. **(A)** HA-modified copolymers are recognized by CD44, which is overexpressed on glioma cells, and are taken up through CD44-mediated endocytosis. **(B)** HA-modified nanomicelles can be administered nasally and rapidly transported to the pontine through the trigeminal pathway, avoiding the BBB and achieving efficient delivery of gliomas. Adapted with permission from reference 110, 112. Copyright 2018 American Chemical Society and 2021 Elsevier B.V., respectively.

Numerous investigations use HA to functionalize NPs for enhanced drug delivery and imaging. For example, HA-modified copolymers are recognized by CD44 receptors on glioma cells. This interaction leads to cellular uptake *in vitro* through mediated endocytosis, as depicted in Figure 5A 110. Once at the target, HA-drug conjugates can also improve the local efficacy of chemotherapy and immunotherapy 111. To circumvent the BBB, other approaches in animal models show that nasally administered HA-modified nanomicelles can be rapidly transported to the brainstem via the trigeminal pathway, achieving efficient delivery to gliomas (Figure 5B) 112. Several reports describe magnetic or metallic NP cores coated with HA to achieve tumor selectivity, stability, and biocompatibility. For instance, one study established superparamagnetic iron oxide NPs (SPIONs) stabilized with an HA coating, which provided excellent colloidal stability, improved biocompatibility, robust GBM cell uptake, *in vitro*, and *T*_2_ contrast potential for MRI 62, 89.

**Figure 6:**
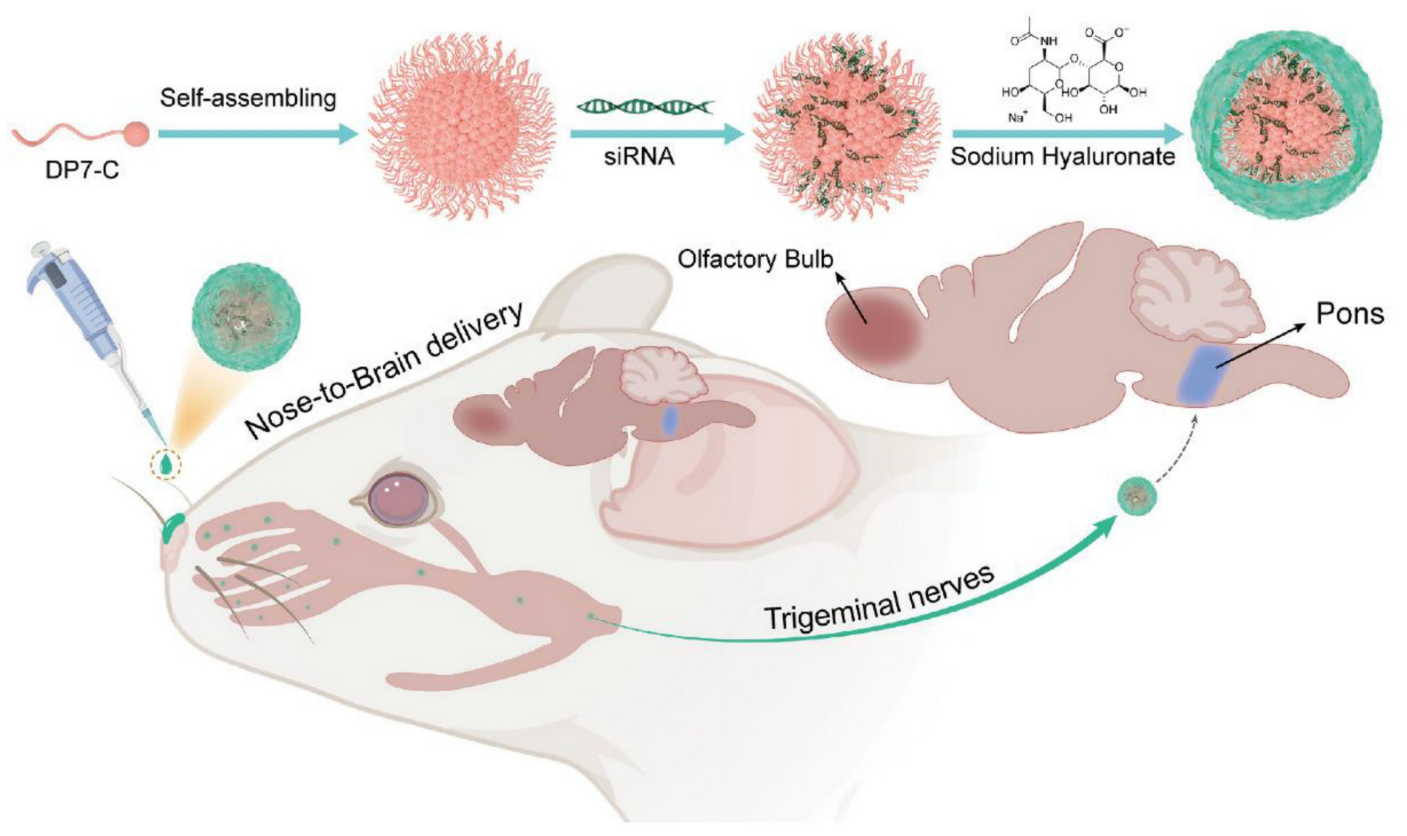
Schematic illustration of hyaluronan-enveloped peptide nanomicelles for the treatment of glioma**.** HA-drug conjugate can improve the efficacy of local chemotherapy and immunotherapy for glioma. Adapted with permission from reference 114. Copyright 2023 Elsevier Ltd.

HA-modified lipid-based systems have also shown promise in GBM therapy by encapsulating or conjugating various payloads. One investigation encapsulated Dox in HA-liposomes to target CD44-overexpressing GBM cells, revealing a marked increase in tumor growth inhibition in an animal model relative to free Dox 113. However, comparative evaluations of HA-systems reveal a "binding site barrier" phenomenon. High-affinity HA nanocarriers often bind irreversibly to CD44 receptors at the tumor periphery, effectively preventing penetration into the hypoxic, necrotic core where resistant stem cells reside. This suggests that while HA enhances tumor accumulation, simply maximizing receptor affinity may be counterproductive; optimizing ligand density to allow for detachment and deeper diffusion is likely required for superior efficacy (Figure 6) 114.

Genetic material delivery via HA coatings further illustrates the scope of these nanotechnologies. HA-grafted lipid NPs were reported to deliver siRNA against Polo-Like Kinase 1, achieving over 80% gene knockdown and significantly prolonged survival in an orthotopic GBM model 114. One group developed HA-lipid NP systems designed to deliver RNA interference (RNAi) constructs directly to GBM cells in culture, achieving favorable uptake and silencing efficiency. Another study used a similar HA-lipid platform to deliver miR-181a, prolonging survival in an *in vivo* tumor model by enhancing accumulation in GBM tissue 115. Overall, HA-functionalized NPs show considerable promise for improving diagnostic accuracy, therapeutic efficacy, and multimodal approaches in GBM management, though further validation is necessary to confirm their *in vivo* stability and safety.

Efforts have also focused on HA-based hydrogels designed to provide localized therapy. Injectable or implantable hydrogel composites formed from HA and other polymers offer sustained drug release *in situ*, aligning mechanical properties with those of brain tissue and improving intracranial drug retention. One group demonstrated CHI/HA/PEI hydrogels that entrapped LLP-DOX, slowing drug release *in vitro* over 148 hours while preserving low genotoxicity 69. Another investigation employed a cucumber uril-crosslinked HA hydrogel with 98% water content, facilitating straightforward placement within resection cavities in a preclinical surgical model and enabling therapeutic antibody delivery 116.

Beyond drug delivery, HA-rich scaffolds are employed to investigate cellular behaviors, including GBM invasion, chemoresistance, and proliferation, in physiologically relevant 3D microenvironments. One report systematically varied HA concentration in a soft matrix and identified in a 3D culture system a biphasic effect on patient-derived GBM invasion, with specific HA thresholds maximizing invasive capacity 117. Parallel findings demonstrated that CD44 binding via HA, together with integrin interactions, can augment resistance to alkylating agents, more accurately mimicking *in vivo* response patterns than conventional sphere cultures 118. Despite the promise of these biomimetic approaches, patient-specific variability in HA-driven invasion emphasizes the need for tailored matrix formulations that capture disease heterogeneity while supporting reproducible screening.

Alternate strategies seek to deplete or remodel HA within the tumor microenvironment instead of incorporating it as a scaffold or coating. One study employed an oncolytic adenovirus encoding soluble hyaluronidase, demonstrating in an animal model that localized HA degradation improved viral dispersion and reduced glioma burden 119. This strategy stands in contrast to formulations that exploit intact HA-CD44 interactions but presents a complementary avenue to counteract the dense ECM barriers limiting drug and gene vector penetration.

Collectively, these findings establish HA as a multifaceted component in the development of next-generation GBM therapies. HA-based NPs leverage CD44 receptor binding to improve drug uptake, provide MRI contrast, and enable synergistic treatments combining photothermal or photodynamic approaches. HA-centered hydrogels and 3D scaffolds can serve as controlled drug depots and physiologically relevant disease models, facilitating localized therapy and elucidating tumor-ECM interactions. In parallel, strategies that degrade HA in the tumor microenvironment attempt to disrupt physical and molecular barriers to treatment, offering a distinct route for enhancing drug penetration. By capitalizing on HA's unique capacity to bind CD44, mimic native ECM, and modulate therapeutic delivery, PSC-based nanomedicines stand poised to expand the therapeutic landscape for GBM 120, 121.

### 4.3 Dextran-based approaches

Dextran, a PSC composed predominantly of α-1,6-glycosidic bonds, has received growing attention in GBM therapy due to its ease of chemical modification (Table 4). Its backbone carries multiple hydroxyl groups that enable flexible conjugation to drugs, antibodies, or targeting molecules, thereby tailoring dextran-based nanosystems for specific therapeutic interventions 122, 123. Such modifications can lessen unwanted plasma protein interactions, prolonging circulation times that heighten drug delivery. The polymer's customizable surfaces help NP evade opsonization and clearance, thus providing sufficient time for transport across the BBB or blood-tumor barrier (BTB) 124, 125. This extended presence elevates drug residence in the bloodstream and subsequent tumor accumulation, particularly where hydrophilic surfaces and versatile chemical derivatization reduce reticuloendothelial uptake 126. By finely tuning NP size and surface properties, these systems can traverse biological barriers selectively, concentrating anticancer agents at the desired site 127, 128. Dextran can be equipped with redox- or pH-sensitive linkers to target intracellular drug release in tumor cells, exploiting high GSH levels or acidic compartments for enhanced specificity 125.

Several groups have harnessed dextran in NP systems alongside other polymers or linkers to advance gene and drug therapy. One formulation hinged on ionic complexation of low molecular weight protamine (LMWP) and dextran sulfate to condense nucleic acids in GBM models 129. These cationic NP demonstrated effective nucleic acid delivery in both two- and 3D cultures, as well as in zebrafish, but detailed validation against human intracranial complexities remains limited. Another dual-sensitive NP employed dendrimer-dextran conjugation via matrix metalloproteinase- and pH-responsive linkers to release DXR *in situ* 130. While this approach improved NP retention and penetration in murine glioma, heterogeneous enzymatic activity could affect uniform disassembly in clinical settings.

In a related strategy, dextran T-10 served as a linker to fuse a single-chain antibody (scFv) recognizing EGFRvIII with DXR, generating an antibody-drug conjugate with enhanced cytotoxicity in EGFRvIII-positive glioma cells 131. Although this design illustrates the impact of receptor-driven targeting, scaling scFv production and ensuring reproducible conjugation chemistry pose nontrivial challenges. Dextran-based NP have also facilitated combination therapies that tackle redundancy in anti-apoptotic pathways. A pH-responsive dextran platform delivered both ABT-263 and an Mcl-1-selective antagonist in orthotopic xenografts, showing superior efficacy while sparing healthy tissue 132. Collectively, these examples reinforce dextran's adaptability for drug or gene vector delivery, particularly when linkers exploit pH or enzymatic triggers to refine release. Future development must reconcile disparities between preclinical models and human disease, including robust tumor microenvironment simulations critical for eventual clinical translation.

Beyond NPs, dextran can be formulated into hydrogels or scaffolds for localized drug delivery in GBM. A dextran-hydroxyethyl methacrylate (dex-HEMA) hydrogel embedded with dexamethasone (DXM) micelles and dexamethasone phosphate liposomes produced a biphasic release *in vitro*, with freely diffusing dexamethasone for up to two weeks before the gel degraded and released the phosphate form 133. This release profile surpasses conventional steroid regimens in sophistication but requires careful crosslink density calibration to accommodate *in vivo* variability. Another injectable system combined Dox-loaded ferritin with oxidized dextran to create Schiff base bonds, gradually discharging intact ferritin particles in an animal model 134. Further expansion of dextran hydrogels includes pH-sensitive designs that encapsulate realgar quantum dots (QDs) and a metabolic inhibitor, achieving synergistic action with radiotherapy in preclinical studies 116. Additionally, acetalated dextran (Ace-DEX) offers tunable degradation rates, allowing controlled local release of agents such as PTX, everolimus, and Dox in tailored scaffolds. Slow-release versions proved valuable for tumor resection sites, whereas faster release was advantageous for micrometastatic disease, though polymer synthesis variability may challenge clinical standardization. These hydrogel and scaffold concepts underscore dextran's versatility for sustained, site-specific therapy in GBM, particularly when coupled with radiosensitizing strategies. Comparative analyses under uniform intracranial models can elucidate the most effective release profiles, but rodent surgical procedures may not capture the full spectrum of human tumor infiltration. Ensuring reproducible polymer manufacturing and precisely tuning drug release are crucial for regulatory viability 135, 136.

Concurrently, iron oxide platforms coated or crosslinked with dextran have gained traction for imaging and combined treatment. Several investigations in animal models have shown that dextran-coated iron oxide NPs can load antisense oligonucleotides, cross the BBB, and accumulate in tumor sites, monitored by MRI and optical methods. Nonetheless, such platforms frequently demand additional surface modifications to maintain colloidal stability and thwart off-target uptake.

Parallel work highlights dextran's potential to enhance imaging resolution and hyperthermia. For instance, a dextran@Fe_3_O_4_ NP formulation improved susceptibility-weighted imaging of glioma vasculature in an animal model 137. Another approach leveraged dextran-coated SPIONs for hyperthermia in animal studies, optimizing dosing regimens to counter organ-level accumulation, though significant uptake still occurred in the spleen, liver, and lungs 138. Dextran-shelled magnetite NPs have also mediated radiosensitization in preclinical models, likely by boosting reactive oxygen species during X-ray exposure, but the enhancement can vary across different radiation modalities 139. In animal models, chitosan-dextran hybrids exhibited improved MRI-based tumor delineation yet faced suboptimal penetration into certain regions of the tumor. Altogether, these findings affirm dextran's stabilizing role, its capacity to improve contrast performance, and its promise for tackling GBM through combination treatments, albeit with persisting hurdles in heterogeneity and off-target distribution. Progressing to large-animal or clinically oriented imaging protocols is paramount for validating dextran-coated platforms in real-world scenarios.

Overall, dextran-based systems—from NP carriers to hydrogel scaffolds and iron oxide platforms—showcase exceptional utility in addressing the multifactorial challenges of GBM. Dextran's adjustable functionalization and capacity for stimulus-sensitive release can collectively reinforce strategies to cross the BBB and concentrate therapeutics at tumor sites. Stimuli-responsive linkers harnessing pH or redox gradients, as well as ligands that promote receptor-mediated uptake, further improve therapeutic efficiency. Despite the promise shown in various *in vivo* models, methodological disparities—ranging from radiation modes to resection techniques—must be harmonized to support clinical integration. Ongoing work should focus on refining large-scale manufacturing, ensuring consistent biodistribution, and clarifying long-term safety before dextran-based approaches can seamlessly transition into standard of care.

### 4.4 Alginate-based approaches

Alginate, derived from the cell walls of marine brown algae, is frequently used in prolonged drug release strategies for GBM. Its linear structure, comprising β-D-mannuronic acid and α-L-guluronic acid residues, undergoes ionic crosslinking and various chemical modifications to yield biocompatible, biodegradable, and minimally immunogenic hydrogels and NP. Because alginate hydrogels can form under physiological conditions, they can fill irregular tumor resection cavities or be injected for localized drug delivery, and their mucoadhesive and bioadhesive properties can improve drug penetration and retention in GBM regions. This PSC's ability to entrap and gradually release therapeutics helps sustain effective drug concentrations in the tumor bed over prolonged intervals, and adjusting the molecular weight and mannuronic-to-guluronic acid ratio can confer pH- or redox-responsiveness. Further functionalizing alginate with specific ligands enables active targeting of glioma cells, relying on receptor overexpression to concentrate drugs in tumors while minimizing collateral damage in healthy tissue. Although some of these approaches await further validation, they illustrate alginate's versatility as a platform material. While primarily utilized for therapeutic delivery and tissue engineering in these studies, its physicochemical properties also support the potential future engineering of combined theranostic systems.

This capacity for controllable gelation and sustained release is particularly beneficial for local GBM treatment, as shown by two complementary investigations. The first study developed a semi-synthetic PSC molecularly imprinted polymer (MIP) hydrogel comprising calcium-crosslinked alginate-poly(N-isopropylacrylamide) graft copolymer, which demonstrated thermo-thickening and shear-thinning behavior with high drug entrapment efficiency (84.59% for ruxolitinib) over a 14-day release period 20. This formulation significantly inhibited GBM cell proliferation (U251 and A172) *in vitro* by reducing colony formation and slowing wound healing, although *in vivo* evidence remains limited. In contrast, a second study employed an injectable alginate-based hydrogel containing a sonosensitizer prodrug and semiconducting polymer NPs, which generated singlet oxygen upon US irradiation and cleaved singlet oxygen-sensitive linkers to release a pathway inhibitor 140. Beyond direct tumor cell destruction, this approach showed potential for triggered, localized treatments in preclinical models. While both hydrogels demonstrate potential efficacy against GBM, variability in matrix composition and mechanisms of activation suggests the need for standardized comparative evaluations.

Several investigations also highlight how alginate scaffolds improve the physiological relevance of GBM models by offering 3D architectures that mirror tumor morphology and microenvironmental cues. One study optimized a 5 wt% gelatin and 5 wt% sodium alginate hydrogel, confirmed by rheological and spectroscopic analyses, to enhance GBM cell viability *in vitro* 141. Within this matrix, tumor spheroids maintained strong proliferative capacity, and administering a CD73 inhibitor reduced VEGF and HIF1-α expression. This sensitivity to targeted therapy underscores the platform's potential for screening new anti-GBM compounds. A parallel 3D co-culture approach used alginate fibers as porogens to incorporate endothelial cells into a PEG hydrogel, enabling spatial compartmentalization that simulates *in vivo* tumor-endothelial interactions 142. Compared to simpler models, this setup promoted GBM cell proliferation while preserving endothelial phenotype *in vitro*.

For larger-scale applications, a microscale alginate tube (AlgTube) system supported long-term culturing of GBM tumor-initiating cells with minimal loss of stem cell markers. This platform sustained approximately 700-fold expansion over 14 days and achieved a high volumetric yield (~3.0 × 10^8 cells/mL). Unlike other 3D methods that may compromise yield over time, the AlgTube approach enabled multiple passages while maintaining essential phenotypes, indicating utility in testing therapies that require large numbers of GBM cells. Taken together, these scaffold-based models better recapitulate tumor-like conditions and offer feasibility for high-throughput experiments, although further validation in organotypic or patient-derived xenograft settings remains necessary 143.

Alginate's versatility also extends to capturing or immobilizing GBM cells in macroporous matrices, which can concentrate therapeutic agents in the tumor area without harming nearby healthy tissue. One study engineered a sodium alginate hydrogel with pore diameters up to 225 μm, showing enhanced capture and retention of F98 GBM cells *in vitro*, especially when functionalized with RGD peptides targeting integrins common in GBM 144. Another investigation employed a similarly macroporous alginate-RGD matrix to sequester F98 cells for irradiation, achieving complete cell eradication within the scaffold *in vitro* while preserving mechanical properties 145. These approaches rely on physical confinement of infiltrative tumor cells, thereby allowing high-dose treatment in confined regions. They differ in methodologies for pore characterization and imaging, yet both illustrate the promise of trapping GBM cells in local matrices. Future research should combine these capture-based platforms with other targeted modalities, examining how pore geometry and biochemical cues affect treatment responses *in vivo*.

### 4.5 Heparin-based approaches

**Figure 7:**
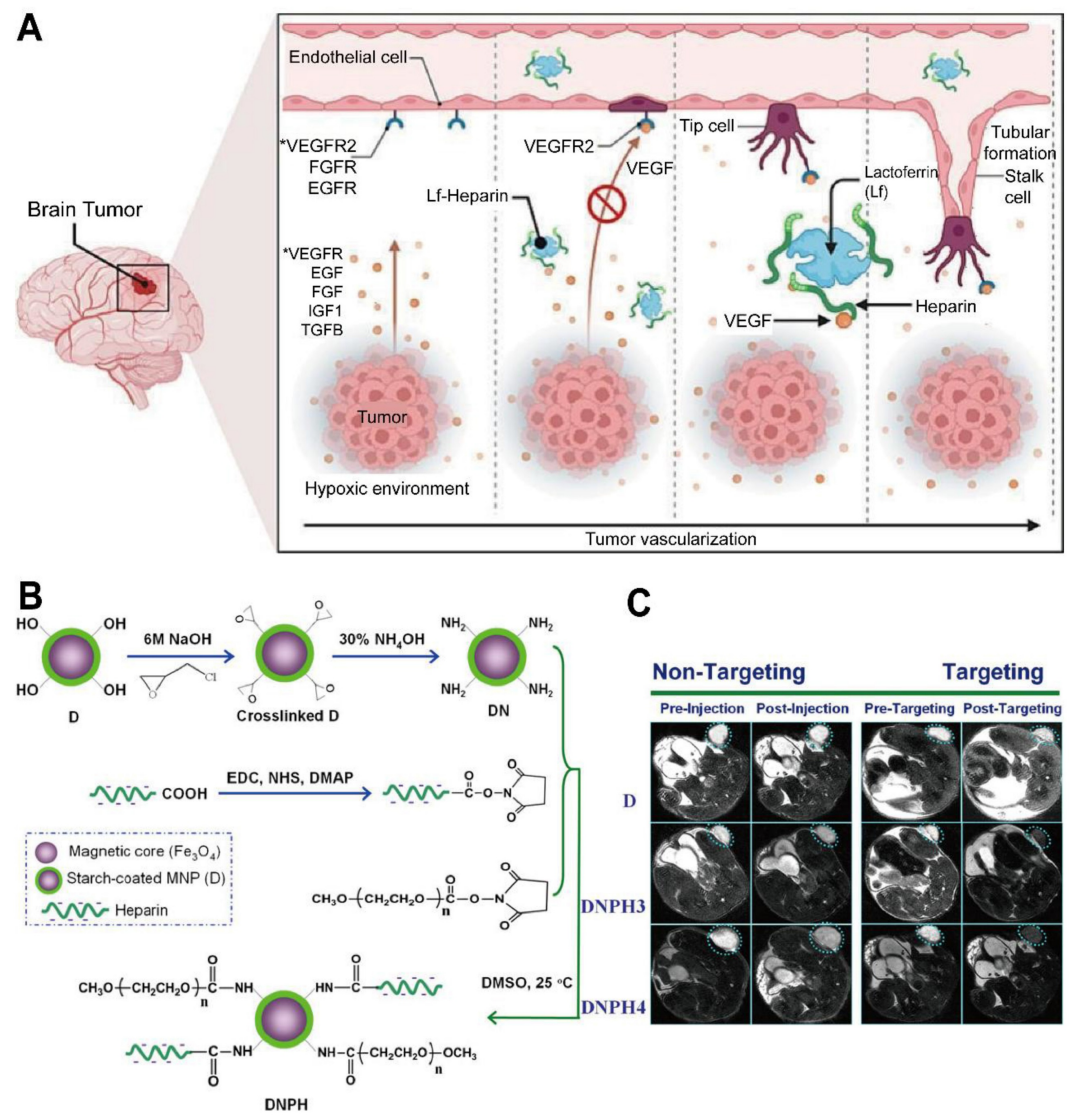
Integrated study of heparin-based nanomaterials in glioma diagnosis and treatment. **(A)** The lactoferrin-heparin coupling is absorbed through the gastrointestinal tract and transported across the BBB to the brain glioma site through transferrin-mediated transport, which limits tumor angiogenesis by disrupting VEGF-VEGFR interactions. **(B)** A novel DNPH for simultaneous MRI and tumor targeting. **(C)** DNPH showed effective enrichment at glioma sites. Adapted with permission from reference 146, 147. Copyright 2023 Elsevier B.V. and 2013 Springer Science+Business Media New York, respectively.

Increasing evidence underscores the potential of heparin-based platforms for GBM therapy by enhancing drug delivery and therapeutic efficacy in GBM. These platforms can serve integrated roles in diagnosis and treatment; for instance, a lactoferrin-heparin (Lf-heparin) complex achieved oral bioavailability and selective transport across the BBB in a preclinical animal model. As illustrated in Figure 7A, this orally administered complex is absorbed in the small intestine and transported across the BBB to the glioma, where it limits tumor angiogenesis by disrupting VEGF-VEGFR interactions 146. This targeted distribution curtailed angiogenic progression and supported less invasive administration routes. Another novel heparin-based nanoparticle (DNPH) was developed for simultaneous magnetic resonance imaging (MRI) and tumor targeting (Figure 7B), showing effective enrichment at glioma sites in an animal model (Figure 7C) 147. For instance, heparin-polyethyleneimine (HPEI) NP were engineered to encapsulate shRNA targeting COUP-TFII, an angiogenic transcription factor in gliomas, regulating tumor vascularization in an orthotopic mouse model 148. The incorporation of heparin facilitated nucleic acid loading while minimizing toxicity. Similarly, researchers synthesized a heparin-taurocholate conjugate (LHT7) to address limitations of unfractionated heparin 149, showing in U87 GBM and human umbilical vein endothelial cells that LHT7 reduced cell viability and endothelial sprouting while suppressing phosphorylation of phospho-ERK and phospho-VEGFR2 150. Another approach integrated a heparin-containing polymer with US-targeted microbubble destruction for the delivery of cilengitide (CGT), resulting in lower renal clearance, enhanced tumor retention of the payload, and extended median survival in a mouse model when combined with US-mediated delivery 151, 152. Collectively, these advances highlight the capacity of PSC conjugates to bolster drug loading, mitigate rapid clearance, and improve tumor specificity. Further refinements may optimize these constructs by adjusting heparin chain length or incorporating additional targeting ligands to accelerate clinical translation.

Beyond serving as a carrier, heparin also affects glioma cell signaling and pathogenetic pathways. At therapeutically relevant concentrations, heparin and its derivatives blocked ectonucleotidase activity by inhibiting extracellular adenosine production in an NPP1 (CD203a)-expressing glioma cell line 153. Conversely, heparin disrupted NP uptake in U87 and GL261 glioma cells in a dose-dependent manner, requiring serum factors for inhibition 154. In a related *in vitro* investigation, heparin-mediated obstruction of exosome traffic diminished the ability of irradiated glioma cell-derived exosomes to promote proliferative advantages in recipient cells 155. These observations suggest a dual role for heparin, as it may safeguard against vesicle-mediated tumor progression but also interfere with nano-delivery strategies. Elucidating precise binding interfaces between heparin and vesicle surfaces could inform more selective interventions, enabling researchers to leverage heparin's biochemical breadth while preserving efficient cargo transfer.

Beyond its use in designing nanocarriers and modulating specific cellular pathways as discussed above, heparin's primary and well-established clinical application is as an anticoagulant. Understanding this systemic role, including its benefits and risks, is crucial when considering any heparin-based therapeutic intervention, especially in the context of GBM, as clinically, GBM patients often face an elevated risk of thrombotic events, prompting explorations of anticoagulant strategies and optimal timing. One retrospective cohort of HGG patients showed that rivaroxaban was associated with a higher incidence of bleeding complications than apixaban and enoxaparin, despite comparable baseline characteristics 156. Another report found that prophylactic heparin administered within 24 hours postoperatively did not significantly increase major bleeding, suggesting early anticoagulation may be feasible 157. In an analysis of atrial fibrillation management in GBM or brain metastasis, anticoagulation did not necessarily escalate intracranial hemorrhage risk 158. A separate series demonstrated encouraging safety and efficacy outcomes for direct oral anticoagulants in GBM patients experiencing postoperative pulmonary embolism, albeit with a need for larger prospective trials 159. A case report highlighted the rare occurrence of heparin-induced thrombocytopenia (HIT) or related cross-reactivity in a glioma patient, requiring high-dose intravenous immunoglobulin and rivaroxaban after fondaparinux proved ineffective 160. These findings underscore the delicate balance between achieving effective thromboprophylaxis and limiting bleeding risk in central nervous system malignancies. Further prospective comparisons of heparin formulations, along with research on predictive biomarkers for bleeding or thrombotic complications, may streamline anticoagulation practices in GBM care.

### 4.6 Cyclodextrin (CD)-based approaches

**Figure 8:**
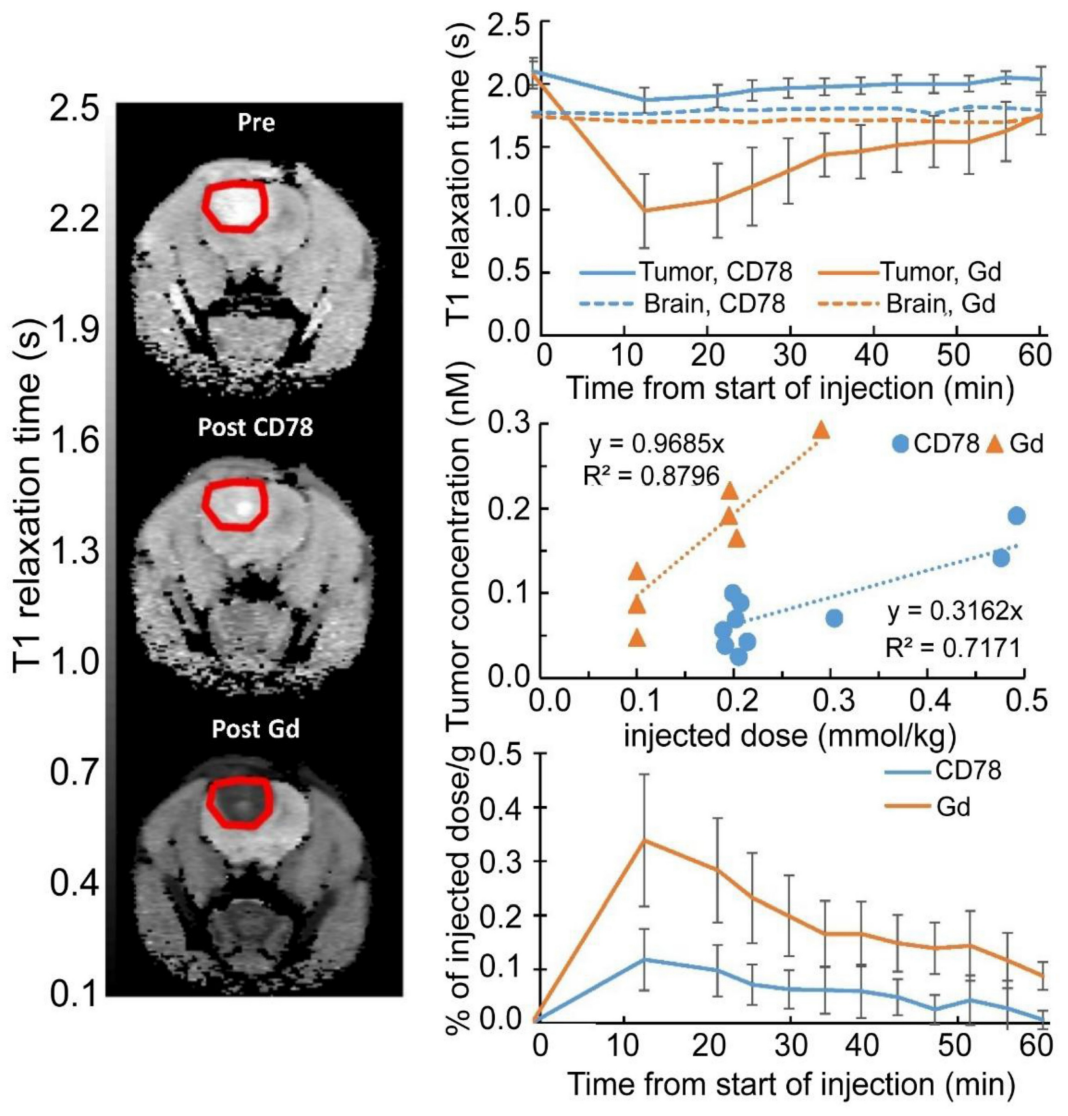
Nonmetallic magnetic resonance contrast agent based on ORCAs as a CD-based carrier for glioma imaging *in vivo*. Adapted with permission from reference 161. Copyright 2020 Wiley-VCH Verlag GmbH & Co. KGaA.

**Figure 9:**
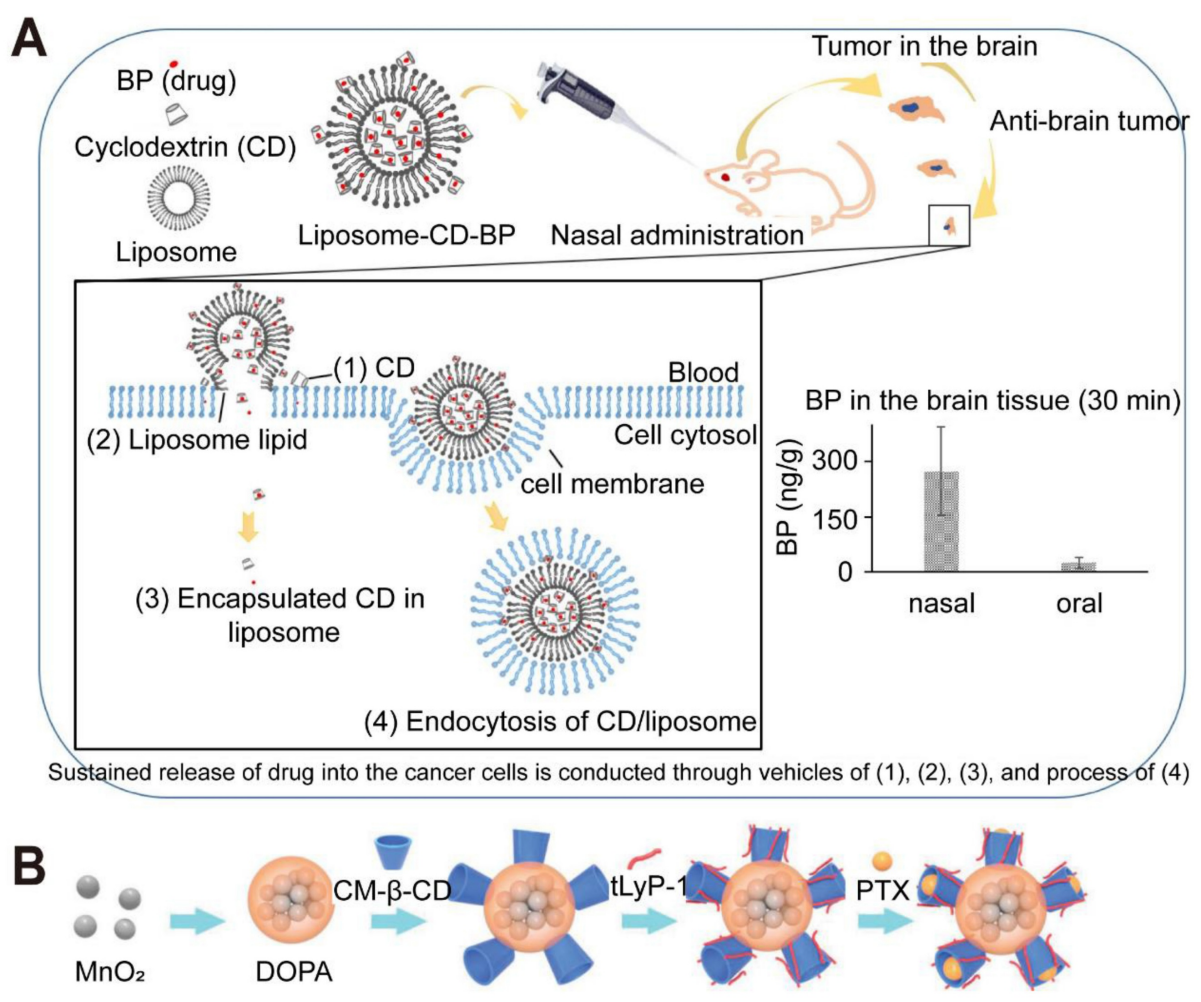
Integrated study of CD-based nanomaterials in the diagnosis and treatment of glioma. **(A)** A CDD1 for non-invasive intranasal administration, a novel carrier system that significantly improves encapsulation efficiency and drug retention time. **(B)** tLyP-1 peptide-modified DOPA-beta-CD-coated PTX and supported MnO_2_ NPs (tLyP-1-CD-DOPA-MnO_2_@PTX) effectively deliver PTX to the glioma site, while producing Mn^2+^ and O_2_ to relieve hypoxia. ROS is produced to kill tumor cells and enhance glioma chemotherapy. Adapted with permission from reference 162, 164. Copyright 2022 Wiley-VCH GmbH, Weinheim, and 2020 by the authors (open access), respectively.

CD-based inclusion complexes alleviate the challenge of poor water solubility often observed in anticancer agents for GBM therapy (Table 5). These nanomaterials serve as integrated platforms for both diagnosis and treatment, evolving from novel water-soluble MRI contrast agents for *in vivo* glioma imaging (Figure 8) 161 to advanced drug delivery vehicles. For instance, peptide-modified NPs coated with cyclodextrin can effectively deliver paclitaxel to the glioma site, relieve tumor hypoxia, and enhance chemotherapy in animal models (Figure 9A) 162. Encapsulating disulfiram in β-cyclodextrin derivatives improves its solubility by approximately 1000-fold, leading to enhanced *in vitro* efficacy against GBM cells (IC50 around 7000 nM), although it remains somewhat less potent than in melanoma cells 163. A similar approach using liposomal carriers enriched with CD, a system designed to improve encapsulation efficiency and drug retention (Figure 9B) 164, increased the retention of butylidenephthalide (BP) in GBM cells for over 8 hours, prolonging median survival in animal models bearing TMZ-resistant tumors. Other studies confirm the versatility of CD encapsulation in various chemical contexts, including a cyclodextrin-calixarene-based amphiphilic system that encapsulates docetaxel and TMZ at over 80% efficiency 165. Moreover, methyl-β-cyclodextrin facilitates perillyl alcohol loading and influences sodium-potassium ATPase-Src signaling in GBM cell lines, despite partial cholesterol depletion complicating mechanistic interpretations 166. CD-based formulations have also proven beneficial for amphiphilic or macromolecular agents: ferrocenyl tamoxifen analogues incorporated into methylated cyclodextrin frameworks retain cytotoxic potency *in vitro* in the nanomolar range 167, while β-cyclodextrin conjugation enhances hydrophobic porphyrins used in photodynamic therapy in preclinical models 168. Although many data derive from *in vitro* or early *in vivo* assays, these findings highlight CD complexes as promising vehicles when transitioning to more advanced glioma models.

In addition to improving solubility, CD constructs offer opportunities to bypass the BBB through intranasal delivery and ligand-mediated targeting. One study employed a β-cyclodextrin-Chitosan hybrid polymer to coat gold-iron oxide NP loaded with therapeutic microRNAs, achieving significant brain accumulation and improved survival in orthotopic GBM-bearing mice when combined with systemic chemotherapy 19, 105. This hybrid formulation serves as a quintessential theranostic model, effectively merging diagnostic capabilities (multimodal MRI/optical imaging) with therapeutic action (miRNA gene regulation) into a single, cohesive entity. Intranasal administration of cyclodextrin-encapsulated BP delivered via liposomes resulted in approximately tenfold higher brain drug levels in animal models compared with oral administration. Further targeting efficiencies emerge when CD is paired with specific ligands: a multifunctional NP containing a magnetic iron oxide core, cyclodextrin, fluorescein, PTX, and chlorotoxin (CTX) enhanced intracellular drug delivery and reduced tumor viability *in vitro* more effectively than the same NP without CTX 69. Conjugating siRNA to β-cyclodextrin similarly improves knockdown activity in cell culture, particularly when targeting ligands are present on the NP surface 169. Although limited sample sizes in some experiments, along with heterogeneity in glioma models, complicate broad conclusions, these intranasal and ligand-targeted approaches offer a valuable route for enhanced drug localization in GBM 170.

CD-based systems also lend themselves to multimodal imaging and diagnostic applications in GBM. For instance, β-cyclodextrin functionalized with piperidine or pyrrolidine structures has demonstrated water-soluble MRI contrast capabilities, exhibiting distinct kinetic stabilities and relaxivities at various field strengths. *In vivo* testing in rats with gliomas documented prolonged *T*_1_ relaxation in the tumor region for at least 60 minutes 171. CD-conjugated iron oxide NP provide parallel opportunities for both imaging and therapeutic payload delivery. Beyond imaging, cyclodextrins serve as building blocks for advanced biosensors: an electrochemical immunosensor platform incorporating graphene oxide, magnetic NPs, and β-cyclodextrin composites directly detected methylated cytosines in the O6-methylguanine-DNA methyltransferase (MGMT) promoter, employing Ru(NH3)63+ as an electrochemical reporter 172. Although further validation in clinically relevant settings is necessary, these developments demonstrate the feasibility of integrating imaging and diagnostic functions with CD-based carriers.

Controlled and stimuli-responsive release is another key benefit conferred by CD-containing materials. Polymeric membranes or hydrogels often entrap drug-CD complexes to regulate release kinetics, as shown by a cationic starch-derivative/poly(vinyl alcohol) membrane loaded with β-cyclodextrin/curcumin, which prolonged cytotoxic activity against GBM cells for up to 96 hours 173. Nanoassemblies that incorporate cyclodextrin-calixarene leverage elevated GSH levels in tumor tissue to disassemble and facilitate on-demand drug delivery in preclinical models. Further strategies use pH-sensitive linkages: adamantane-modified DXR conjugated to cyclodextrin-based polymers via hydrazone bonds remains stable under neutral conditions but releases drug in acidic environments 174. High-affinity binding approaches, such as divinyl sulfone cross-linking in β-cyclodextrin 175 or incorporating antiangiogenic compounds for slow elution 176, underscore the adaptability of CD carriers. Directly conjugating siRNA to cyclodextrin similarly hinges on balanced stability and release, with targeting ligands enabling more efficient intracellular uptake *in vitro* 169. These polymeric approaches still require careful optimization of crosslink density and cleavage kinetics, but they reveal considerable potential for sustained, localized dosing of GBM treatments 177.

Collectively, these lines of research highlight the capacity of CD constructs to improve solubility, bioavailability, targeting, imaging, and controlled release in GBM therapy. Although differences in experimental designs, cell models, and dosing protocols have produced some variations in outcome, a consistent underlying principle emerges: cyclodextrin-based systems can integrate multiple functionalities to enhance therapeutic efficacy against GBM in preclinical models. Ongoing studies seeking to optimize CD formulations through standardized procedures and expanded *in vivo* evaluation will likely accelerate their clinical translation as promising treatment strategies for this challenging disease.

### 4.7 Other PSCs

PSC-based biomaterial scaffolds have gained prominence in GBM management by leveraging their biocompatibility and adjustable physicochemical features. Multiple studies investigated the potential of pectin hydrogels, which are plant-derived PSCs with variable esterification levels that can be ionically crosslinked with calcium to form robust 3D matrices. One investigation prepared low-esterified pectin films via ionic gelation, culminating in NP measuring 90-115 nm with negative zeta potentials between -8.30 and -7.86 mV 178. These NP significantly inhibited U87MG human GBM cells, potentially by modifying cell adhesion, yet demonstrated no toxicity towards non-tumor cells. Another approach utilized sprayable pectin-based hydrogels loaded with polylactic acid-PEG nanocrystals, generating localized anticancer drug delivery in *ex vivo* brain models 179. In addition, including ECM proteins such as collagen modulates glioma cell morphology. One report showed that pectin hydrogels incorporating different collagen I/IV ratios influenced C6 glioma cell growth patterns and neurite formation 180, while pectin fractions derived from Campomanesia xanthocarpa induced 15.55-37.65% cytotoxicity in human GBM cells and heightened ROS levels without harming normal cells 181. These observations align with a related study in which collagen films were functionalized with chondroitin sulfate to enhance U87 GBM cell adhesion and proliferation 182, 183. Despite these promising effects, much of the efficacy data derive from *in vitro* or limited *ex vivo* models with small sample sizes or single tumor cell lines. Nonetheless, the collective evidence suggests that pectin-based biomaterials offer customizable physicochemical properties suitable for glioma cell inhibition or targeted drug transport. Future research will likely focus on refining *in vivo* delivery platforms or implantable systems capable of synchronizing local ECM composition with controlled drug release.

While the aforementioned PSC-based biomaterials offer promise as therapeutic delivery systems and modulators of the tumor environment, attention must also be given to the inherent roles of specific proteoglycans within the glioma milieu, which themselves can be pivotal therapeutic targets. In this regard, research on chondroitin sulfate proteoglycans (CSPGs) emphasizes their crucial role in shaping glioma cell behaviors. Multiple studies have identified elevated levels of chondroitin sulfate proteoglycan 4 (CSPG4) and associated enzymes in high-grade gliomas, where they contribute to tumor maintenance, stemness, and therapy resistance. One study reported that CSPG4 and its glycan chains are essential for glioma-initiating cell (GIC) self-renewal, and their removal triggers GIC differentiation 184. Mathematical modeling further revealed that CSPG-rich environments facilitate noninvasive tumor growth linked to reactive astrocyte encapsulation, while reduced chondroitin sulfate expression correlates with a more invasive phenotype 185. In addition, the increased expression of chondroitin sulfate biosynthetic enzymes, such as CHSY1 (chondroitin sulfate synthase 1), predicts poor prognosis, partially through stabilizing PDGFRA and bolstering PDGF-induced signaling 186, 187. Elevated chondroitin sulfate content is often detected near necrotic and perivascular regions of GBM tumors and is associated with heightened proliferation 188. Targeting CSPG4 with immunotoxins substantially improved cytotoxicity *in vitro*, especially when combined with Bcl-2 inhibitors, underscoring the clinical potential of CSPG4-focused therapies 189. However, large-scale antibody production and heterogeneity pose challenges for translating these therapies to clinical use 190. Despite methodological limitations, including small patient cohorts and preclinical models, these findings converge to indicate that CSPG4 and the machineries driving aberrant chondroitin sulfate expression serve as attractive therapeutic targets by sustaining critical survival signals within the tumor microenvironment.

Conventional GBM treatments can unintentionally reshape the glycosylated ECM, influencing subsequent tumor regrowth or infiltration 191. One study in adult Wistar rats demonstrated that extended TMZ therapy reduced chitosan levels in brain tissue by 1.5- to 2.5-fold, in part through downregulating aggrecan core protein, possibly contributing to increased anxiety despite an absence of overt histological damage 192. Combined TMZ and DXM regimens also modified CSPGs in healthy brain regions, leading to enhanced adhesion and proliferation of GBM cells in organotypic slice models 193. Ionizing radiation (IR) further disrupts proteoglycan synthesis, with triple irradiation (7 Gy over three days) in an animal model downregulating decorin, biglycan, versican, and brevican, alongside decreases in chondroitin sulfate and heparan sulfate (HS) 194. Glioma cells cultured with irradiated tissue exhibited intensified adherence and proliferation, indicating that IR-induced glycan remodeling may accelerate tumor recurrence. Although these studies employed small animal cohorts and had limited follow-up, they underscore the need to consider unplanned ECM alterations when administering chemoradiation therapies. Further investigation may clarify whether interventions that maintain healthy chondroitin sulfate profiles can mitigate these unintended effects and slow tumor regeneration.

In parallel with scaffold-based strategies, efforts to disrupt glycosaminoglycan (GAG) biosynthesis have gained momentum as a means of dismantling tumor-promoting ECM elements 195. One investigation developed small-molecule inhibitors targeting xylosyltransferase (XYLT-1) and β-1,4-galactosyltransferase-7 (β-GALT-7), which catalyze early steps of GAG chain assembly. Using hydrophobic prodrugs bearing 4-deoxy-4-fluoro-2,3-dibenzoyl-xyloside cores, researchers achieved submicromolar to micromolar IC50 values in U251 and U87 GBM lines, presumably by halting GAG elongation. Although these findings suggest that inhibiting GAG biosynthesis can abrogate malignancy-related signaling, challenges persist in delivering these inhibitors across the BBB and avoiding off-target effects, given the importance of GAG pathways in normal tissues. This approach may benefit from combination therapies incorporating GAG inhibition with biomaterial scaffolds that sequester growth factors and precisely deliver anticancer agents. Further *in vivo* validation could facilitate the integration of GAG inhibitors into broad anticancer regimens while minimizing toxicity to non-tumoral cells.

Collectively, current research on other PSCs in GBM underscores the versatility of PSC-based nanomedicines, scaffold technologies, and ECM-directed therapies. Pectin scaffolds, alone or combined with collagen and chitosan, show promise for local drug delivery and tumor growth modulation, while evidence linking CSPGs to stemness and therapeutic resistance highlights the need to target chitosan pathways. Standard chemotherapy and radiotherapy can inadvertently weaken the ECM through GAG depletion, potentially promoting GBM relapse. Pharmacological inhibition of GAG biosynthesis represents a complementary strategy, as small molecules that interfere with glycan-chain elongation may temper the survival signals maintained by aberrant chitosan expression. Ongoing research integrating scaffold design, targeting of CSPGs, and GAG biosynthesis blockade holds considerable potential to refine GBM treatment and ultimately improve clinical outcomes.

## 5. Immunotherapeutic Applications of PSC-Based Nanomedicines

PSC-based nanomedicines address key GBM immunotherapy limitations—specifically poor antigen presentation and the immunosuppressive microenvironment, by delivering immunomodulators and leveraging intrinsic immunostimulatory properties (e.g., Chitosan, β-glucans) 5. Specifically, by enhancing the delivery of tumor-specific cargos to antigen-presenting cells (APCs) and concurrently activating innate immunity, these carriers address critical challenges such as poor antigen presentation and insufficient immune cell activation. Nanoconstructs based on chitosan, HA, or botanical PSCs are being engineered to improve dendritic cell (DC) maturation, boost cytotoxic T lymphocyte (CTL) responses, and favorably modulate cytokine profiles towards pro-inflammatory signals. For instance, dextran can be used for conjugating immunostimulants, and its derivatives can facilitate dual delivery of drugs and immune mediators 81. HA nanocarriers are often used to co-deliver chemotherapeutics alongside immunologic adjuvants, bolstering immune-mediated antitumor effects. These actions collectively work to dismantle barriers to T cell infiltration, showing promise in transforming GBM from an immunologically 'cold' tumor to one that is responsive to immune-mediated clearance.

To address challenges in delivering and sustaining immunotherapeutic effects within the GBM microenvironment, PSC-based hydrogels offer solutions for localized and prolonged activity. For instance, an injectable oxidized high-amylose starch hydrogel was developed to overcome the difficulty of effectively delivering and supporting immune cells locally. By serving as a scaffold for macrophages and co-delivering polarizing agents, this hydrogel bypassed the BBB and fostered a supportive niche that modulated macrophage behavior towards an anti-tumor phenotype, thereby boosting therapeutic efficacy 196. This system tackled the issue of poor immune cell infiltration and survival by providing an interconnected porous structure. Similarly, to counteract the rapid clearance and systemic toxicity associated with conventional chemo-immunotherapy, a chemically crosslinked chitosan hydrogel was engineered for sustained local co-delivery of doxorubicin (DXR) and an immune checkpoint inhibitor (BMS-1). This approach solved the problem of insufficient drug retention in GBM lesions, achieving prolonged local release and significantly enhancing chemo-immunotherapy outcomes by preventing uncontrolled polymer swelling and maintaining therapeutic concentrations 197. Chitosan's utility in chemo-immunotherapy is further exemplified by systems where Dox-loaded bovine serum albumin NPs crosslinked with chitosan alongside anti-PD-1 checkpoint inhibition prompt immunogenic cell death and stimulate a durable anti-tumor immune response 100. Dextran-based hydrogels can also be coupled with immunomodulatory strategies; for example, an injectable system combining Dox-loaded ferritin with oxidized dextran gradually discharged intact ferritin particles, promoting antitumor responses, and has been explored with immune checkpoint inhibitors. A remaining challenge for such platforms is optimizing the hydrogel design to ensure the long-term viability and functional efficacy of embedded immune cells or labile immunomodulators, requiring a delicate balance between mechanical integrity and nutrient diffusion.

PSC scaffolds are also being engineered to solve issues of suboptimal immune stimulation by conventional immunotherapies. For example, to overcome the poor delivery and systemic toxicity of STING (Stimulator of Interferon Genes) agonists, a HA-STING agonist conjugate (HA-MSA2) was developed. This local polymer-drug delivery system effectively bolstered immunogenic cell death and STING-related cytokine production, thereby addressing the challenge of inadequate innate immune activation and successfully reshaping the immune microenvironment by recruiting NK and CD8+ T cells in murine GBM models 198. Another strategy to tackle insufficient tumor immunogenicity and promote T cell responses involves a chitosan-based hydrogel. This system, by co-delivering DXR-loaded bovine serum albumin NPs with anti-PD-1 checkpoint blockade, addressed the need for sustained induction of immunogenic cell death and broader immune activation. The prolonged DXR release, coupled with chitosan's intrinsic immunostimulatory properties, effectively promoted tumor-specific T cell responses 100. The versatility of HA-based systems extends to their use with immunostimulatory agents. For instance, cationic polyethylenimine coated with selenocysteine and sodium hyaluronate has been combined with natural killer cells to cross the BBB and exhibit potent GBM cytotoxicity, highlighting the potential of HA for multimodal immunotherapeutic approaches. HA-based NPs can enable synergistic treatments combining photothermal, photodynamic, or immunotherapeutic approaches 112. Such PSC-based approaches demonstrate the capacity to overcome distinct hurdles in immunotherapy, with the HA-STING system targeting specific pathway activation and the DXR-chitosan design offering multi-faceted immune engagement.

Further innovations in PSC hydrogels aim to address the critical challenges of off-target toxicity and insufficient spatiotemporal control in GBM immunotherapy. An alginate-based injectable prodrug hydrogel (APN) employing sonodynamic therapy (SDT)-responsive NPs was designed to tackle these issues by enabling on-demand release of an IDO inhibitor, a key immunosuppressive pathway target 142. Ultrasound-triggered singlet oxygen generation not only provoked immunogenic cell death but also cleaved oxygen-sensitive linkers to release the IDO (immunosuppressive pathway) inhibitor precisely at the tumor site. This strategy directly addresses the problem of systemic exposure and non-specific immunomodulation, although optimizing ultrasound penetration in brain tissue remains a hurdle. Another approach to overcome the limitations of systemic toxicity and achieve potent, localized immune activation involves HA-drug conjugates. For instance, an HA matrix co-delivering DXR and the Toll-like receptor-9 agonist CpG successfully induced robust immunogenic cell death and CD8+ T cell responses at reduced drug dosages, thereby mitigating systemic side effects often seen with free-drug administrations 199. Despite these delivery successes, a recurring reason for therapeutic failure in PSC-based immunotherapy is the profound immunosuppression of the GBM microenvironment, which nanocarriers alone cannot always reverse. While carriers like alginate or HA successfully deliver immune agonists, they do not inherently alter the physical stiffness of the extracellular matrix (ECM) that physically excludes T-cells. Consequently, inconsistent findings across studies often correlate with the density of the tumor ECM, indicating that delivery efficiency does not linearly translate to immune activation in "cold" GBM tumors.

Alginate formulations have also shown the capacity to reprogram macrophages or T cells within the glioma microenvironment, and engineered alginate NP or hydrogels can exhibit efficient macrophage uptake, modulating immune cell function for combined chemo-immunotherapy approaches 200. Future research on alginate-based capture platforms should explore combinations with immunotherapies, examining how pore geometry and biochemical cues shape immune responses *in vivo*. Collectively, these PSC hydrogel systems demonstrate significant potential in solving key problems in GBM immunotherapy: they function as localized immunomodulatory reservoirs that ensure controlled drug release, enhance immune activation specifically at the tumor site, and reduce systemic toxicity, potentially improving patient compliance. Future work will focus on refining hydrogel properties to further sustain immunotherapeutic action and minimize physical impacts on brain tissue, thereby advancing therapeutic precision for GBM.

Beyond hydrogels, PSC nanoparticle platforms offer distinct advantages for tackling immunotherapy challenges in GBM. Their inherent tunability allows for the incorporation of responsive elements (e.g., pH-, enzyme-, or redox-sensitivity) that can solve problems related to non-specific drug release and off-target effects of potent immunomodulators 178. Dextran NPs, for example, can leverage intrinsic immunostimulatory effects. Dextran derivatives can be engineered to amplify or modulate immune responses, supporting combination therapies geared toward eliminating malignant cells and invigorating antitumor immunity. Dextran's immunomodulatory capability complements cytotoxic regimens designed to overcome the immunosuppressive tumor milieu. Although unmodified dextran has low immunogenicity, specialized derivatives can prompt beneficial immune activation by enhancing antigen presentation 125. Such systems can deliver antigens or immune-stimulating molecules to antigen-presenting cells in glioma, strengthening T cell infiltration and function and fortifying the immunosurveillance network 201. Dextran can also consolidate immunogenic cell death in tandem with DAMPs released during chemotherapy. Combining triggered release approaches with immunotherapeutic elements boosts dextran's versatility; immunostimulatory ligands, adjuvants, or monoclonal antibodies can be conjugated or co-encapsulated to stimulate dendritic cell maturation and augment cytotoxic T cell expansion. By pairing targeted therapy with immune activation, dextran-based formulations address resistance mechanisms and dampen immunosuppression, notably through M2-to-M1 macrophage polarization. Key design refinements in PSC NPs are crucial for enhancing immunotherapeutic efficacy. For instance, controlling particle size can address the poor penetration of immunotherapies into deep tumor tissues, while appending immunostimulatory ligands directly onto NP surfaces can solve the problem of insufficient local immune activation. Such modifications aim to ensure that immunotherapeutic payloads are delivered precisely to immune cells or the tumor microenvironment, thereby amplifying desired anti-tumor immune responses. By strategically designing PSC NPs to overcome specific hurdles in GBM immunotherapy, such as poor bioavailability of immune agents or inadequate engagement of immune effector mechanisms, these systems hold the potential to significantly improve treatment outcomes 202, 203. Rigorous preclinical and clinical validation will be crucial to translate these sophisticated NP designs into effective GBM immunotherapies.

Beyond their role as delivery vehicles, certain PSCs possess inherent immunomodulatory properties that can be harnessed to directly address the immunosuppressive nature of GBM. Heparins, for example, tackle the problem of adenosine-mediated immunosuppression by allosterically inhibiting the ectonucleotidase CD203a. This action limits extracellular adenosine accumulation (thereby reducing immunosuppressive effects) and curtails the development of immunosuppressive regulatory T cell (Treg) phenotypes, thus synergizing with T cell-based immunotherapies 153. Heparin-based NPs have also been studied for their role in regulating tumor vascularization and immunosuppression. A significant challenge, however, is heparin's systemic anticoagulant activity, which complicates its therapeutic application. PSC-based formulation strategies, such as controlled-release systems or structural modifications to heparin itself, are being explored to solve this issue by segregating its tumor-specific immunomodulatory benefits from its systemic effects on coagulation. Investigating these refined approaches is key to unlocking heparin's clinical potential in GBM immunotherapy.

Fungal β-glucans exemplify another category of PSCs with intrinsic immunostimulatory capabilities, offering a means to counteract immune coldness in the GBM microenvironment. These agents have been shown to activate microglia, leading to the secretion of pro-inflammatory cytokines that inhibit tumor proliferation and promote apoptosis 204. However, their application faces immunotherapy-specific challenges, including the translational complexities of *ex vivo* microglial conditioning and the poor BBB penetration of high molecular weight β-glucans, which restricts their efficacy upon direct administration. To overcome these limitations, PSC-based formulation strategies, particularly nanoparticle encapsulation, are being investigated. Such approaches aim to solve the problems of targeted delivery and BBB traversal, thereby enhancing the therapeutic window and reducing dosage requirements for β-glucans. These efforts underscore the utility of leveraging both the intrinsic immunomodulatory effects of PSCs and advanced delivery systems to augment their impact, especially in combination with other immunotherapies like checkpoint inhibitors.

In conclusion, PSC-based nanomedicines and inherently immunomodulatory PSCs offer multifaceted solutions to critical challenges hampering effective immunotherapy for GBM. Whether by acting as sophisticated carriers that overcome delivery barriers and improve the targeting of immunostimulatory agents, or by exerting direct immunomodulatory effects, these biopolymers are pivotal in enhancing immune system engagement against brain tumors 203, 204. Strategies employing materials like heparin and β-glucans demonstrate how PSCs can directly counteract specific immunosuppressive mechanisms within the GBM microenvironment. Dextran's mild immunostimulatory properties can enhance immune responses, and ongoing research explores combining dextran scaffolds with immunotherapies. As immuno-oncology evolves, dextran's chemical modifiability may expand its role in uniting targeted drug delivery with robust immune activation. Similarly, the immunotherapeutic targeting of chondroitin sulfate proteoglycans (CSPGs) is an active area of research for other PSC-based strategies. The success of these approaches hinges on innovative engineering to solve inherent limitations, such as systemic side effects (e.g., anticoagulation) or poor bioavailability (e.g., restricted BBB penetration). By focusing on how PSC-based systems can resolve existing problems in immunotherapy—such as poor drug localization, insufficient immune activation, significant off-target toxicities, and the immunosuppressive tumor niche—research is paving the way for transformative treatment regimens. Continued refinement of their biochemical properties and delivery mechanisms promises to shift the balance in GBM from immune evasion towards a pro-inflammatory state amenable to robust and sustained anti-tumor immunity.

## 6. Main Challenges for PSC-Based Nanomedicines Applied Clinically

Despite the extensive library of preclinical successes described in this review, the translation of PSC-based nanomedicines to the clinic remains stagnant. Currently, no PSC-specific nanocarriers have received FDA approval for Glioblastoma, and extremely few have advanced to Phase I/II trials, in stark contrast to the clinical success of lipid-based nanoparticles (e.g., Onpattro® or COVID-19 vaccines). This translational gap is not due to a lack of efficacy, but rather the unique barriers imposed by natural polymer sourcing. A fundamental barrier to clinical progression is the challenge of ensuring manufacturing reproducibility and achieving Good Manufacturing Practice (GMP) compliance 205. The transition of multifunctional PSC nanoplatforms from bench-scale synthesis to industrial-scale production is hampered by the complex, often bottom-up processes required for creating theranostic systems 206. PSCs sourced from natural materials often vary in molecular weight, branching structure, and purity due to environmental factors, which introduces raw material variability. Unlike synthetic polymers (e.g., PLGA) which have defined stoichiometries, natural PSCs like chitosan and alginate suffer from batch-to-batch inconsistencies driven by seasonal and species-specific variations in the raw source material (e.g., crustacean shells or algae type). Furthermore, a critical but often overlooked barrier is endotoxin (lipopolysaccharide) contamination. Cationic PSCs bind avidly to negatively charged endotoxins, making purification notoriously difficult. This is a "deal-breaker" for clinical translation, as the regulatory limit for endotoxins in intrathecal/intracranial products is significantly stricter than for intravenous drugs to prevent aseptic meningitis.

This, combined with complex synthesis strategies, can lead to significant batch-to-batch inconsistencies in critical quality attributes such as particle size, surface charge, and drug-loading capacity 207. This variability is particularly problematic for multi-functional PSCs (e.g., those combining targeting ligands, drugs, and imaging agents). The stochastic nature of chemical conjugation often results in "polydisperse" batches where some nanoparticles are heavily targeted while others are naked. This heterogeneity complicates the establishment of the structure-activity relationships required by regulatory bodies like the FDA, representing a primary bottleneck preventing these promising academic studies from entering Phase I trials. Such variability can drastically affect stability, biodistribution, and therapeutic performance, hindering the comparability of preclinical and clinical data 208. To overcome these challenges, robust engineering solutions are essential. Standardized synthesis protocols, supported by automated quality control measures, are crucial for minimizing variability. Furthermore, modular manufacturing platforms, such as microfluidic synthesis, are increasingly being adopted to allow for the precise, continuous, and highly reproducible fabrication of NPs, often maintaining superior control over particle size compared to traditional bulk methods 209. Simplifying the chemical synthesis pathways for complex carriers also enhances the potential for large-scale, cost-effective GMP production.

A related challenge is the scalability and consistency of ligand conjugation, which is necessary to achieve specific targeting and successful BBB penetration 210. The attachment of targeting moieties (e.g., peptides, antibodies, aptamers) must be highly efficient and reproducible without compromising nanoparticle integrity or biological activity 211. Given the high structural complexity and functional variability of GBM tumors, where receptor expression is heterogeneous, robust and adaptable conjugation methods are required 212. Efficient bio-orthogonal chemistries, such as copper-free click reactions, offer an attractive solution, as they enable the precise incorporation of targeting ligands onto nanoparticle surfaces in a manner that is both reproducible and scalable 213. Research also indicates that tuning the density and affinity of targeting ligands—with intermediate affinities often yielding enhanced BBB transport—must be consistently maintained throughout the manufacturing scale-up process to guarantee predictable biological outcomes 214.

Furthermore, the path from promising preclinical investigation to approved GBM treatments is significantly obstructed by regulatory hurdles. Agencies such as the U.S. Food and Drug Administration (FDA) and the European Medicines Agency (EMA) impose rigorous demands for comprehensive safety and efficacy data, including thorough evaluations of pharmacokinetics (PK), biodistribution, potential toxicity, and immunogenicity. A critical challenge in this domain is bridging the "preclinical translation gap." While rodent models are essential for initial screening, their physiological differences from humans often lead to poor prediction of safety and efficacy. Consequently, there is a growing regulatory expectation for robust preclinical studies in large animal models, such as canines or non-human primates, particularly for CNS-targeted nanomedicine. These models provide more relevant data on complex pharmacology, neuro-toxicology, and immunogenic responses due to their closer similarities in brain structure, immune systems, and metabolic pathways. However, conducting such extensive pharmacology and toxicology studies is resource-intensive, ethically complex, and presents significant logistical challenges, thereby slowing the translational pipeline. This scrutiny is particularly intense for novel nanomaterials targeting the central nervous system (CNS), where off-target effects and long-term accumulation pose unique neurotoxicity risks 215, 216. Regulatory considerations for CNS delivery go beyond standard toxicology. Agencies require specialized neurotoxicology assessments, specifically monitoring for CNS inflammation (gliosis), brain edema, and lowering of the seizure threshold—adverse events that standard systemic toxicity studies often miss. Additionally, demonstrating the stability of the PSC-nanocarrier complex in cerebrospinal fluid (CSF), which has a different ionic strength and protein composition than plasma, is a mandatory but frequently neglected step in the Investigational New Drug (IND) enabling process. Although naturally derived PSCs generally exhibit low immunogenicity, the complex chemical modifications required for theranostic functionality—including PEGylation, cationization, or ligand integration—can inadvertently alter their biological identity and toxicological profiles, potentially triggering unforeseen immune responses or enhancing clearance by the reticuloendothelial system (RES). To mitigate these risks and meet regulatory thresholds, strategies include shifting towards biodegradable materials, such as specific PSC derivatives and lipid-based formulations, which offer improved clearance profiles. The development of "self-destructing" nanocarriers that degrade upon drug delivery is also being explored to reduce the potential for long-term tissue retention, addressing a primary safety concern for CNS applications 217.

Finally, the vast, high-dimensional design space of PSC-based nanomaterials presents a fundamental challenge that traditional, low-throughput experimental methods cannot efficiently address. This is where AI-driven discovery and design represent a new frontier. The optimization of a nanocarrier for GBM involves a complex interplay of variables, including polymer source, molecular weight, branching, chemical modifications, ligand density, and particle morphology. Machine learning (ML) models, trained on emerging datasets, can accelerate the prediction of structure-activity relationships (SAR) and critical quality attributes. These models can screen virtual libraries of PSC derivatives to identify novel materials with optimized properties for BBB penetration, tumor-specific targeting, and payload delivery. Furthermore, generative AI approaches may enable the de novo design of bespoke PSC-based nanostructures with precisely tailored theranostic functions. The primary hurdle, however, lies in the generation and curation of large, high-quality, and standardized datasets required to train reliable predictive models—a task compounded by the very manufacturing inconsistencies that plague the field. Integrating "closed-loop" systems, which combine AI-driven design with automated, high-throughput synthesis platforms (like microfluidics), is a formidable but necessary challenge to accelerate the rational design and clinical translation of next-generation PSC nanomaterials for GBM.

## 7. Conclusions

In summary, PSC-based nanoplatforms possess excellent biocompatibility, inherent stimuli-responsiveness, and versatile chemical modifiability. These properties underpin promising advancements, but realizing their full clinical potential requires a structured and visionary approach. The future perspective for engineering PSC nanoplatforms in GBM (GBM) theranostics centers on overcoming key translational challenges—specifically, bypassing the formidable BBB and navigating tumor heterogeneity—through the synergistic integration of advanced methodologies. This new generation of multifunctional nanoplatforms must be intelligent, precisely targeted, and capable of personalized adaptation. The convergence of bio-orthogonal chemistry, artificial intelligence (AI)-driven design, and patient-derived models is poised to revolutionize the rational development, preclinical testing, and clinical readiness of PSC-based delivery systems.

A crucial opportunity lies in leveraging bio-orthogonal chemistry for enhanced nanoplatform functionalization and targeted delivery. Bio-orthogonal reactions, such as the Inverse Electron-Demand Diels-Alder (IEDDA) reaction or copper-free click chemistry, enable the highly specific and rapid conjugation of targeting ligands (e.g., peptides or antibodies), therapeutic payloads, or imaging agents onto PSC carriers. Crucially, this occurs under mild physiological conditions. This precision is vital for directing nanoplatforms toward GBM cells or stromal components, thereby enhancing specificity toward the tumor microenvironment (TME), minimizing off-target effects, and overcoming the long-standing challenge of stabilizing conjugations without diminishing bioactivity. Furthermore, these chemical tools facilitate advanced strategies, such as pre-targeting, where a modified PSC carrier first accumulates at the tumor site, followed by a second component that rapidly "clicks" into place, enhancing local drug activation. Bio-orthogonal strategies can also be employed for cellular engineering, allowing the introduction of artificial receptors onto immune or stem cells to create highly specific cellular vehicles that guide nanoplatforms directly to the tumor.

The complexity inherent in optimizing nanoplatform design—including composition, size, morphology, surface charge, and stimuli-responsive drug release kinetics—necessitates the adoption of AI-driven strategies. Machine learning (ML) and deep learning algorithms, such as convolutional neural networks (CNNs), are becoming indispensable for predicting optimal synthesis parameters, which can accelerate the development cycle, reduce costs, and refine large-scale manufacturing. Computational modeling and AI can be utilized to predict the binding affinity of nanocarriers to specific receptor targets, forecast BBB permeability, and assess potential toxicity (ADMET parameters) *in silico* before laboratory synthesis. Beyond nanocarrier optimization, AI-enabled platforms are crucial for analyzing vast data sets from multi-omics profiling and radiomics, enabling the stratification of GBM patients and the customization of nanomedicine compositions to match individual tumor molecular signatures. AI-assisted algorithms are also essential for optimizing the material properties of PSC-based biomaterials, predicting how variations in structure influence therapeutic efficacy.

To ensure translational success, these intelligently designed and precisely functionalized nanoplatforms must be rigorously validated in human-relevant preclinical models. Patient-derived models (PDMs), including GBM organoids (GBOs), patient-derived cancer organoids (PDCOs), and advanced microfluidic BBB chips, are crucial for this purpose. Unlike traditional two-dimensional cultures, GBOs and PDCOs retain the histopathological, genomic, and molecular heterogeneity of the original tumor and facilitate the study of TME interactions. The integration of GBOs into organ-on-a-chip (OOC) devices, particularly those incorporating perfusable, patient-specific microvascular systems, is promising. These devices provide an accurate platform for high-throughput screening to assess nanoplatform transport across the BBB and evaluate patient-specific treatment responses. Moving forward, the fusion of PDM technology with AI will allow for timely analysis of treatment response, generating predictive digital twins that simulate therapeutic futures and accelerate the clinical pipeline for highly customized PSC nanotheranostics.

Finally, the versatility of PSC nanoplatforms makes them ideal vehicles for synergistic combination therapies. Certain PSCs can intrinsically activate immune cells, suggesting powerful opportunities to combine immunomodulators, adjuvants, or cytokines with chemotherapy for synergistic effects. The integration of PSC nanoplatforms with radiotherapy could also enhance treatment outcomes by co-delivering radiosensitizers. Moreover, these carriers are promising for advanced genetic medicine; co-delivery of gene-editing tools, such as CRISPR-Cas9 plasmids or siRNA, could disrupt oncogenes, although ensuring high specificity in GBM cells remains a formidable challenge that may be addressed by the targeting strategies discussed above.

In conclusion, addressing the multifaceted challenges in production, standardization, and safety is critical for clinical translation. Aligning research methods and establishing uniform testing conditions using the advanced models described will be vital for moving PSC-based approaches from the laboratory to the clinic. With continued innovation and cooperation among academia, industry, and regulators, the intelligent design and application of PSC-based nanomedicines hold considerable promise as a future therapeutic modality for GBM.

## Figures and Tables

**Table 1:** PSC-Metal Hybrid Imaging Probes

Approach/Method Name	Nanoparticle Composition	Targeting Moiety	Imaging Modality & Contrast	Therapeutic Agent	Validation Model	Authors
Folic Acid & Hyaluronic Acid Coated SPIONs	SPION core; Hyaluronic acid & Folic acid shell	Hyaluronic acid; Folic acid	MRI (*T*_2_)	Intrinsic cytotoxicity	Glioma & adenocarcinoma cells; hepatocytes	Kasprzyk *et al.* 62
Dextran-Coated SPIONs for RNA Delivery	Iron oxide core; Dextran & Cy5.5 shell	Passive (size-based)	MRI; Optical (Cy5.5)	Antisense oligonucleotide (anti-miR10b)	Orthotopic GBM mouse model	Kim *et al.* 26
Chitosan-Dextran Hybrid SPIONs (CS-DX-SPIONs)	SPION core; Chitosan-dextran hybrid shell	Chitosan (charge-mediated)	MRI (*T*_2_)	None (potential carrier)	U87, C6, HeLa cells; Orthotopic C6 glioma rat model	Shevtsov *et al.* 66
Magnetic Ternary Nanohybrid (MTN) for MSC Transfection	SPION core; Hyaluronic acid & cationic polymer shell	Hyaluronic acid & Magnetic force (for MSCs)	MRI	Gene delivery (TRAIL plasmid) to MSCs	Human MSCs; U87MG orthotopic xenograft model	Huang *et al.* 63
Quantum Dot-Biopolymer-Drug Nanohybrids (ZnS@CMC-DOX)	ZnS quantum dot core; Carboxymethylcellulose shell	None	Optical (fluorescence)	Doxorubicin	GBM & healthy cells	Mansur *et al.* 70
Amino Acid Modified PSC-Capped QDs	AgInS2 quantum dot core; CMCel-L-cysteine or -Poly-L-arginine shell	L-cysteine/poly-L-arginine	Optical (fluorescence)	None	Glioma cells	Carvalho *et al.* 71
Chlorotoxin-Conjugated Magnetic NPs (IONP-PTX-CTX-FL)	Iron oxide core; PEG, Cyclodextrin, CTX, Fluorescein shell	Chlorotoxin	MRI; Optical (fluorescein)	Paclitaxel	GBM & drug-resistant GBM cells	Mu *et al.* 69
Mechanism Study of Poly-l-lysine Mediated MNP Uptake	Magnetic NP core; Dextran or Poly-l-lysine shell	Poly-l-lysine	N/A	None	Human glioma & HeLa cells	Siow *et al.* 68
Low-Molecular-Weight Hyaluronic Acid-SPIONs (Theranostic)	Fe_3_O_4_ core; Low-MW hyaluronic acid shell	Low-MW Hyaluronic acid	MRI (*T*_2_^*^)	Intrinsic cytotoxicity (34% inhibition)	U87MG GBM & NIH3T3 fibroblast cells	Chang *et al.* 64
Low-Molecular-Weight Hyaluronic Acid-SPIONs (Diagnostic)	Fe_3_O_4_ core; Low-MW hyaluronic acid shell	Low-MW Hyaluronic acid	MRI (*T*_2_^*^)	None	U87MG GBM & NIH3T3 fibroblast cells	Huang *et al.* 65

**Table 2:** Chitosan-Based Approaches

Approach/Method Name	Carrier System	Payload/Active Agent	Targeting/Delivery Strategy	Key Finding/Performance Metric	Validation Model	Authors
MTX-Chitosan-HPMCP NPs	Chitosan-HPMCP NPs	Methotrexate (MTX)	P-gp efflux inhibition; Mucoadhesion	Enhanced cytotoxicity (IC50 = 68.79 vs 80.54 µg/mL for free MTX)	U251MG GBM cells	Naves *et al.* 84
iNSC-laden Injectable Chitosan Hydrogel	Injectable thermo-responsive hydrogel	Induced neural stem cells (iNSCs)	Post-surgical local cell delivery	50% increase in median survival vs iNSCs alone	Post-surgical GBM mouse model	King *et al.* 97
Magnetic GO/Chitosan/Iron Oxide Microspheres	Graphene oxide/chitosan/iron oxide microspheres	Temozolomide (TMZ)	Magnetic field- and pH-sensitive release	Drug release doubled in 90 min with 100 Hz magnetic field	GBM cells (MTT assay)	Ahmadi *et al.* 16
AT101-conjugated Chitosan Nanobubbles	Chitosan nanobubbles (NBs)	Delivery platform (unloaded)	GPC1 protein targeting via AT101 antibody	Increased specific tumor delivery compared to unconjugated NBs (p=0.02)	U-87 MG xenograft model	Cintio *et al.* 93
Macroporous Alginate-Chitosan Hydrogel	Macroporous alginate-chitosan hydrogel	Cell trap (no drug)	Physical trapping of GBM cells for subsequent radiotherapy	F98 GBM cells accumulate and are retained within the gel matrix	F98 GBM cells	Parès *et al.* 99
TPP-conjugated Chitosan NPs	Triphenylphosphonium (TPP+)-conjugated chitosan NPs	Temozolomide (TMZ)	Mitochondrial targeting via TPP+; Intranasal delivery	Entrapment efficiency of 93.59%; Greater nasal mucosal retention	Goat nasal mucosa (*ex vivo*); *In vitro* cell lines	Dahifale *et al.* 91
Chemo-Immunotherapy Crosslinked Hydrogel	Pluronic F-127/Chitosan thermo-responsive hydrogel	Doxorubicin (DOX), BMS-1	Intratumoral injection for synergistic chemo-immunotherapy	Tumor 43 times smaller than untreated group	GBM tumor-bearing mouse model	Chuang *et al.* 191
Etoposide-loaded Chitosomes	Chitosomes (chitosan-coated liposomes)	Etoposide	Parenteral administration; Enhanced stability	Heightened efficacy against the U373 GBM cell line	U373 cell line	Gonzalo *et al.* 85
CRT-functionalized Chitosan/HA Nano-emulsion	Chitosan/hyaluronic acid layered nano-emulsion	Paclitaxel	CRT peptide targeting of transferrin receptor on BBB	41.5% higher uptake in brain endothelium cells than negative control	bEnd.3 cells (BBB model)	Capua *et al.* 94
DOX-loaded LLPs in Chitosan/HA/PEI Hydrogel	Liposome-like particles in Chitosan/HA/PEI hydrogel	Doxorubicin (DOX)	Controlled local delivery in resection cavity	Sustained drug release up to 148 hours	GBM spheroids; *In vitro* cell studies	Adiguzel *et al.* 98
Gemcitabine Chitosan-coated PLGA NPs	Chitosan-coated PLGA NPs	Gemcitabine (GEM)	Intranasal delivery; Mucoadhesion	Promoted GEM antiproliferative activity and sensitized cells to TMZ	U215 and T98G human GBM cell lines	Ramalho *et al.* 103
GRP-conjugated Magnetic Graphene Oxide	Chitosan-coated magnetic graphene oxide (mGOC)	Doxorubicin (DOX)	Dual active (GRP peptide) and magnetic targeting	Best potency to suppress tumor growth and prolong animal survival	Orthotopic U87 brain tumor model in mice	Dash *et al.* 27
DXR-loaded Self-crosslinked Hydrogel (DXR-CBGel)	BSA NPs self-crosslinked with chitosan	Doxorubicin (DXR), anti-PD-1 antibody	Localized chemoimmunotherapy as an *in situ* vaccine	Effectively inhibited cancer recurrence post-surgery	GBM lesions (animal model)	Long *et al.* 83
Review of Polymeric NPs for GBM	Chitosan, PLGA, PEG, etc. NPs	Various chemotherapeutics	Active targeting with ligands (transferrin, chlorotoxin, etc.)	Review article summarizing multiple strategies	N/A (Review)	Paula *et al.* 92
3D Chitosan-Hyaluronic Acid Scaffolds	Chitosan-hyaluronic acid (CHA) porous scaffold	3D tumor model (no drug)	High-throughput screening (HTS) platform	Produced uniform response (CV < 0.15) and wide screening window (Z' > 0.5)	Three human GBM cell lines	Zhou *et al.* 108
Curcumin-Chitosan Nano-complex	Curcumin-chitosan nano-complex	Curcumin	Epigenetic modification via gene expression regulation	Significantly increased MEG3 and decreased DNMT gene expression	GBM cell line	Abolfathi *et al.* 106
Chitosan-functionalized Silicon NPs	Chitosan-coated Silicon NPs (SiNPs)	Silicon NPs (photosensitizer)	Passive targeting via Enhanced Permeability and Retention (EPR) effect	Tumor accumulation increased to 39.55% after 7 days	Nude mice with subcutaneous human GBM	Baati *et al.* 88
Sialic Acid-functionalized Selenium NPs@Chitosan	Sialic acid-coated, chitosan-stabilized Se NPs	Selenium NPs (Se NPs)	Surface modification with sialic acid for improved stability/activity	Dose- and time-dependent inhibitory effects on T98 GBM cells	T98 and A172 GBM cell lines	Abadi *et al.* 95
TMZ-loaded Chitosan-based Thermogels	Chitosan-β-glycerophosphate thermogel with SiO2/PCL microparticles	Temozolomide (TMZ)	Local delivery into post-resection cavity	Caused a significant reduction in the growth of tumor recurrences	GBM resection and recurrence mouse model	Gherardini *et al.* 109

**Table 3:** Hyaluronic Acid-Based Approaches

Approach/Method Name	HA-based Core Structure	Therapeutic/Diagnostic Payload	Mechanism/Function	Additional Targeting Moiety	Validation Model	Authors
HA-SPION Dual-Targeted System	Superparamagnetic Iron Oxide Nanoparticle (SPION)	SPION (MRI contrast)	Dual-targeted diagnosis and therapy	Folic acid	Glioma/adenocarcinoma cells (*in vitro*)	Kasprzyk *et al.* 62
CHI/HA/PEI Hydrogel with LLP-DOX	Hydrogel (Chitosan/HA/PEI)	Doxorubicin in liposomes	Local, controlled drug release	N/A (local delivery)	GBM spheroids (3D *in vitro*)	Adiguzel *et al.* 98
IR780-rGO-HA/DOX Nanoplatform	Reduced Graphene Oxide (rGO)	Doxorubicin; IR780 (photosensitizer)	Multimodal therapy (Chemo/PTT/PDT)	N/A	U87 cells; Xenograft tumor model (*in vivo*)	Dash *et al.* 111
ICOVIR17-MSC in sECM	Synthetic ECM (targets endogenous HA)	Oncolytic virus expressing hyaluronidase	Oncolytic virotherapy; ECM degradation	N/A	GBM resection mouse model; PDX xenografts	Martinez *et al.* 119
RGD-Functionalized HA 3D Scaffold	Biomaterial scaffold	N/A (model system)	3D *in vitro* model for studying chemoresistance	RGD peptide (Integrin targeting)	Patient-derived GBM cells	Xiao *et al.* 118

**Table 4:** Dextran-Based Approaches

Approach/Method Name	Dextran Formulation	Payload/Active Agent	Primary Function	Validation Model(s)	Key Innovation	Authors
Dextran Sulfate/LMWP NPs	Ionic complex of dextran sulfate and low molecular weight protamine	Nucleic acids	Non-viral gene delivery	GBM cell lines, 3D spheroids, zebrafish models	Cationic polymeric NPs for gene delivery with negligible toxicity and efficient internalization	Esteban *et al.* 130
Dexamethasone-loaded dex-HEMA Hydrogel	Dextran-hydroxyethyl methacrylate (dex-HEMA) hydrogel	Dexamethasone (micelles) & dexamethasone phosphate (liposomes)	Localized, prolonged anti-edema drug delivery	*In vitro* release kinetics studies	Biphasic and sequential drug release from an immobilized dual-nanoparticle hydrogel system	Straten *et al.* 133
Dextran-Coated Iron Oxide NPs (MN-anti-miR10b)	Crosslinked dextran coating on iron oxide NPs	Antisense oligonucleotide (anti-miR10b), Cy5.5 dye	Image-guided RNA delivery	Orthotopic GBM mouse model	Dual-modal (MRI/optical) tracking of a nano-platform delivering RNAi payload across the BBB	Kim *et al.* 26
PD0721-DOX Antibody-Drug Conjugate	Dextran T-10 as a linker for conjugation	Doxorubicin (DOX)	Targeted chemotherapy (ADC)	EGFRvIII-positive and negative GBM cell lines	Dextran linker to create an scFv antibody-drug conjugate targeting EGFRvIII-expressing cells	Hu *et al.* 131
Nano-realgar Hydrogel (NRA@DH Gel)	pH-sensitive dextran hydrogel with hyaluronic acid coating	Nano-realgar quantum dots (NRA QDs)	Synergistic chemo-radiotherapy	*In situ* GL261 GBM mouse model	Hydrogel acts as a sustainable ROS generator to deplete GSH and enhance radiotherapy efficacy	Wang *et al.* 135
Biomimetic Dextran NPs	pH-sensitive dextran nanoparticle core	ABT-263 and A-1210477 (Bcl-2/Mcl-1 inhibitors)	Brain-targeted combination drug delivery	GBM cell lines, patient-derived cells, orthotopic GBM mouse model	Biomimetic (RBC membrane, ApoE peptide) coating for BBB penetration and synergistic drug delivery	He *et al.* 132
Chitosan-Dextran SPIONs (CS-DX-SPIONs)	Hybrid chitosan-dextran coating on SPIONs	SPIONs (contrast agent)	MRI contrast enhancement & targeted delivery	Glioma cell lines, orthotopic C6 glioma rat model	Hybrid chitosan-dextran coating enhances cellular uptake and tumor retention for improved MRI contrast	Shevtsov *et al.* 66
Acetalated Dextran (Ace-DEX) Fibrous Scaffolds	Acetalated dextran biodegradable fibrous implant	Paclitaxel and Everolimus	Interstitial combination chemotherapy	GBM cell lines, orthotopic GBM resection/recurrence mouse models	Synchronized interstitial release of synergistic drugs from scaffolds with tailored degradation rates	Graham-Gurysh *et al.* 136
Tunable Acetalated Dextran (Ace-DEX) Scaffolds	Acetalated dextran nanofibrous scaffolds	Paclitaxel	Interstitial therapy with controlled release	Orthotopic GBM resection/recurrence and distant metastasis mouse models	Demonstrating that drug release rate is a critical parameter for efficacy in different tumor models	Graham-Gurysh *et al.* 137

**Table 5:** Cyclodextrin-Based approaches

Approach/Method Name	Cyclodextrin Type	Payload/Guest Molecule	Application/Goal	Validation Model	Key Outcome/Performance	Authors
Disulfiram Encapsulation for Drug Repositioning	HP-β-CD, RAMEB, SBE-β-CD	Disulfiram	Enhance drug solubility and stability for cancer therapy	*In vitro* (melanoma, GBM cell lines)	~1000-fold drug solubility enhancement; IC50 ~100 nM on melanoma	Benkő *et al.* 158
Curcumin-Loaded Polymeric Membrane	β-CD	Curcumin	Local drug delivery system for melanoma and GBM	*In vitro* (melanoma, GBM cell lines)	Prolonged cytotoxic effect up to 96 h at 50 μg/mL	Gularte *et al.* 172
Multifunctional Magnetic NPs	Cyclodextrin	Paclitaxel (PTX)	Targeted therapy for drug-resistant GBM	*In vitro* (GBM and drug-resistant GBM cells)	Enhanced cellular uptake and improved efficacy of PTX in GBM cells	Mu *et al.* 69
Cyclodextrin-Encapsulated Liposomes	Cyclodextrin	Butylidenephthalide (BP)	Intranasal delivery for drug-resistant brain tumors	*In vivo* (nude mice with TMZ-resistant GBM)	Encapsulation efficiency up to 95%; 10-fold higher drug accumulation vs. oral	Lin *et al.* 164
Cyclodextrin-siRNA Conjugates	β-CD	siRNA (luciferase, PLK1)	Targeted siRNA delivery for gene silencing	*In vitro* (U87, PC3, DU145 cancer cell lines)	Enhanced gene knockdown achieved with ligand-targeted nanoparticle formulations	Malhotra *et al.* 167
Electrochemical Immunosensor	β-CD (on GO-Fe_3_O_4_ nanocomposite)	Anti-5-methylcytosine (5mC) antibody	Detection of MGMT gene methylation for GBM diagnosis	Biological samples (methylated MGMT-DNA)	Detection limit of 0.0825 pM	Yang *et al.* 170
pH-Sensitive Affinity-Based Delivery System	Cyclodextrin polymer	Adamantane-modified doxorubicin	Tumor-specific, pH-triggered local drug delivery	*In vitro* (U-87 GBM cells)	Sustained release over 87 days with accelerated release at low pH	Cyphert *et al.* 173
